# Differential Effects of Combined ATR/WEE1 Inhibition in Cancer Cells

**DOI:** 10.3390/cancers13153790

**Published:** 2021-07-28

**Authors:** Gro Elise Rødland, Sissel Hauge, Grete Hasvold, Lilli T. E. Bay, Tine T. H. Raabe, Mrinal Joel, Randi G. Syljuåsen

**Affiliations:** Department of Radiation Biology, Institute for Cancer Research, Norwegian Radium Hospital, Oslo University Hospital, N-0379 Oslo, Norway; grroed@rr-research.no (G.E.R.); hausis@rr-research.no (S.H.); grete.hasvold@farmasi.uio.no (G.H.); lilbay@rr-research.no (L.T.E.B.); tinraa@rr-research.no (T.T.H.R.); mrinaljoel@gmail.com (M.J.)

**Keywords:** WEE1 kinase, ATR kinase, replication stress, synergy, combination therapy, lung cancer, radiosensitization, CDK activity

## Abstract

**Simple Summary:**

Cancer cells often show elevated replication stress and loss of cell cycle checkpoints. The ataxia telangiectasia and Rad3-related (ATR) and WEE1 kinases play roles in protecting cancer cells from high replication stress and in regulating the remaining cell cycle checkpoints. Inhibitors of ATR or WEE1 therefore have the potential to selectively kill cancer cells and are currently being tested in clinical trials. However, more studies are needed to understand how these inhibitors work in various types of cancer and to find the most effective ways of using them. Here, we have explored whether simultaneous treatment with ATR and WEE1 inhibitors is a promising approach. Effects were investigated in cell lines from osteosarcoma and lung cancer. We expect our results to be of importance for future treatment strategies with these inhibitors.

**Abstract:**

Inhibitors of WEE1 and ATR kinases are considered promising for cancer treatment, either as monotherapy or in combination with chemo- or radiotherapy. Here, we addressed whether simultaneous inhibition of WEE1 and ATR might be advantageous. Effects of the WEE1 inhibitor MK1775 and ATR inhibitor VE822 were investigated in U2OS osteosarcoma cells and in four lung cancer cell lines, H460, A549, H1975, and SW900, with different sensitivities to the WEE1 inhibitor. Despite the differences in cytotoxic effects, the WEE1 inhibitor reduced the inhibitory phosphorylation of CDK, leading to increased CDK activity accompanied by ATR activation in all cell lines. However, combining ATR inhibition with WEE1 inhibition could not fully compensate for cell resistance to the WEE1 inhibitor and reduced cell viability to a variable extent. The decreased cell viability upon the combined treatment correlated with a synergistic induction of DNA damage in S-phase in U2OS cells but not in the lung cancer cells. Moreover, less synergy was found between ATR and WEE1 inhibitors upon co-treatment with radiation, suggesting that single inhibitors may be preferable together with radiotherapy. Altogether, our results support that combining WEE1 and ATR inhibitors may be beneficial for cancer treatment in some cases, but also highlight that the effects vary between cancer cell lines.

## 1. Introduction

The ataxia telangiectasia and Rad3-related (ATR) and WEE1 kinases are two key players in DNA damage and replication stress response, and both kinases are considered promising targets for cancer treatment [1]. When activated, ATR leads to cell cycle checkpoint arrest in S- and G2-phases, DNA damage repair, replication fork protection, and reduced origin firing [2]. Thus, cells with reduced ATR function will have compromised checkpoints and increased sensitivity to DNA damage and replication stress [3,4]. WEE1 is an important cell cycle regulator and is required for cell cycle checkpoint arrest in S- and G2-phases [5,6]. WEE1 suppresses the activity of cyclin-dependent kinases 1 and 2 (CDK1/2) by mediating the inhibitory phosphorylation on tyrosine 15 [7,8]. WEE1 inhibition therefore causes premature mitotic entry and abrogation of the DNA-damage-induced G2 checkpoint, often resulting in mitotic catastrophe [9]. Furthermore, S-phase WEE1 inhibition causes unscheduled firing of replication origins and activates endonucleases such as MUS81, which can lead to massive DNA damage and replication catastrophe [5,10,11,12].

Inhibitors of ATR and WEE1 are promising as new treatment agents, and recent studies suggest that simultaneous inhibition of ATR and WEE1 might be more beneficial than either single treatment. Firstly, pointing toward a likely synergy between WEE1 and ATR inhibitors, several previous studies have shown synergistic effects on cancer cell killing when the WEE1 inhibitor MK1775 is combined with inhibitors of CHK1, a major downstream effector of ATR [13,14,15,16,17]. We recently found that this synergy can be explained by a synergistic induction of S-phase DNA damage, likely due to WEE1 and CHK1 regulating two different steps in replication initiation, namely CDK activity and CDC45 loading onto chromatin [12]. In another study, focusing on the triple combination of WEE1 and CHK1 inhibitors with gemcitabine, WEE1 inhibition was found to show distinct effects on mitosis not shared by CHK1 inhibition [18]. Furthermore, a few recent studies have also reported synergistic effects upon combining ATR and WEE1 inhibitors [19,20,21,22]. More specifically, WEE1 and ATR inhibitors caused the synergistic killing of acute myeloid leukemia (AML), biliary tract, and breast cancer cells, likely due to problems arising in S-phase and G2/M [19,20,21,23]. Notably, this combination appears to be well tolerated in mouse models, as it showed little toxicity in normal tissue [19]. However, the effects of combined WEE1 and ATR inhibition in other cancer cell types and the mechanisms underlying the synergistic killing remain poorly understood.

Here, we report the effects of the WEE1 inhibitor MK1775 (AZD1775/Adavosertib) and the ATR inhibitor VE882 (VX-970/M6620/Berzosertib), alone and in combination, on the osteosarcoma cell line U2OS and on a panel of four different lung cancer cell lines with different sensitivities to the WEE1 inhibitor. We found a large synergistic reduction in cell viability upon the combined treatment in U2OS and in two of the lung cancer cell lines. However, the combined treatment could not fully compensate for cell resistance to the WEE1 inhibitor. Our findings support that combining WEE1 and ATR inhibitors may be a promising treatment strategy, but more studies will be needed to be able to predict which patients will benefit from such a treatment.

## 2. Results

### 2.1. Combined Inhibition of WEE1 and ATR Gives Synergistic Induction of DNA Damage in S-Phase Accompanied by Synergistic Cell Killing in U2OS Cells

**Figure 1:**
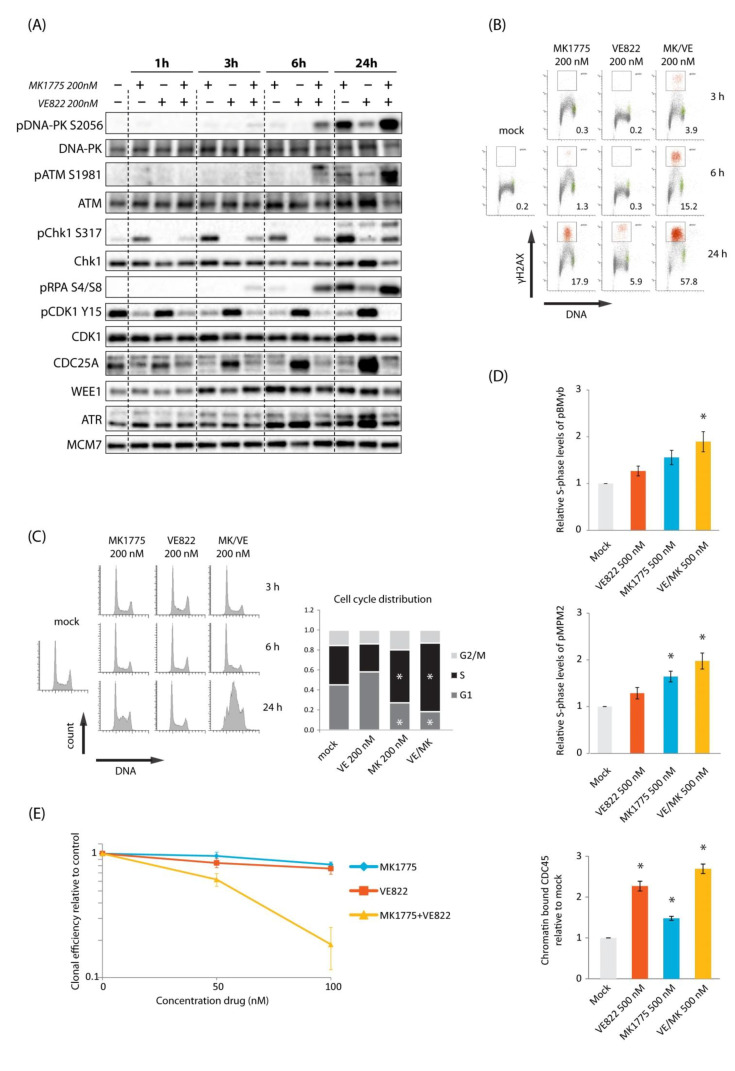
Synergistic induction of S-phase DNA damage and cell killing in U2OS cells upon combined WEE1/ATR inhibition. (**A**) Immunoblotting of extracts from U2OS cells treated with MK1775 (200 nM) and VE822 (200 nM) as indicated. (**B**) Flow cytometry analysis of U2OS cells treated with MK1775 (200 nM) and/or VE822 (200 nM) for 3, 6, and 24 h or left untreated (mock). Scatter plots of γH2AX versus DNA content (Hoechst) are shown. Numbers indicate percentage of cells with strong γH2AX levels (indicated in red color). (**C**) Left: Histograms showing cell cycle profiles (counts versus DNA content) from the same experiment as in (**B**). Right: Quantification of cell cycle distribution from three independent experiments treated as in (**B**) for 24 h. All other results in (**A**–**C**) are representative of three or more independent similar experiments. (**D**) Flow cytometry analysis of phospho-B-MYB, phospho-MPM2, and chromatin-bound CDC45 in U2OS treated with MK1775 (500 nM) and/or VE822 (500 nM) for 1 h. Median levels were measured within a region of cells with S-phase DNA content as indicated in Figures S1D and S3. (**E**) Clonogenic survival of U2OS cells treated with MK1775 and/or VE822 at the indicated concentrations for 24 h. Clonal efficiency relative to that of untreated cells (control) is shown. Error bars: SEM (*n* = 3). In (**C**), *p* values were determined by the two-tailed two-sample Student’s *t* test (test criterion: treated sample ≠ mock), and in (**D**), *p* values were determined by the two-tailed Student’s one-sample *t* test (test criterion: fold change ≠ 1), * *p* ≤ 0.05.

In a previous screen identifying drugs that synergized with the WEE1 inhibitor MK1775 to cause DNA damage in S-phase, we found two CHK1 inhibitors (AZD7762 and LY2603618) to be among the top hits [12]. Since CHK1 is a downstream target of ATR, and ATR is the apical kinase in the replication stress response, we hypothesized that combining MK1775 with an inhibitor of ATR would give a response similar to, or even better than, the combined WEE1/CHK1 inhibition. First, we investigated DNA damage signaling via immunoblotting of U2OS cells, as this cell line was used in our previous experiments on WEE1/CHK1 inhibition [12]. The ATR or WEE1 inhibitors alone (200 nM VE822 or 200 nM MK1775) showed no phosphorylation of DNA damage markers ATM S1981, DNA-PK S2056, or RPA S4/S8 at 1–6 h after treatment, but some phosphorylation occurred at 24 h and was most pronounced with the WEE1 inhibitor (Figure 1A). However, combined treatment with ATR and WEE1 inhibitors caused phosphorylation of these targets already at 6 h and a further increase at later time points, consistent with increased DNA damage (Figure 1A). Similar results were obtained with a different ATR inhibitor (AZD6738) and with siRNA-mediated depletion of WEE1 (Figure S1A,B). Phosphorylation of CHK1 S317, a downstream target of ATR, was induced at all times after treatment with the WEE1 inhibitor alone (Figure 1A). This finding is consistent with previous reports [5,20], and activated ATR likely contributes to suppress induction of DNA damage after WEE1 inhibition. Furthermore, as expected, the WEE1 inhibitor caused reduced levels of CDK1-Y15 inhibitory phosphorylation (Figure 1A), and the ATR inhibitor caused elevated levels of CDC25A in agreement with lack of CDC25A degradation when the ATR/CHK1 pathway is inhibited [24,25].

To study the damage response in individual cells and correlate it with cell cycle effects, we performed flow cytometry analysis of the DNA damage marker γH2AX and cell cycle distribution. In cells treated with the WEE1 inhibitor alone, the whole S-phase population showed a small elevation in γH2AX signals at 3 h and a fraction of cells (~18%) showed strong γH2AX signals at 24 h (Figure 1B). This was accompanied by an accumulation of cells in S-phase at 24 h (Figure 1C, bottom left histogram), indicating high replication stress and problems with S-phase progression. In contrast, no S-phase accumulation was observed in ATR inhibition alone, and only a low fraction of cells (~6%) showed strong γH2AX signals at 24 h (Figure 1B,C). The combined treatment clearly induced synergistic effects, with markedly more cells (~58%) showing strong γH2AX signals at 24 h (Figure 1B), together with a strong S-phase accumulation (Figure 1C). The percentage of cells positive for the mitotic marker phospho-H3, however, was not higher than 5% or 6% at any of the time points after the combined treatment (Figures S1C and S2C, left (U2OS)), indicating no major synergistic effects of the combination of these inhibitors on premature mitotic entry.

A likely cause for DNA damage in S-phase in response to WEE1 and ATR inhibition is increased replication initiation. Consistent with this, we observed elevated CDK activity, as measured via flow cytometry analysis of phospho-B-MYB and phospho-MPM2, and more loading of the replication initiation factor CDC45 in individual S-phase cells 1 h after combined treatment (Figure 1D and Figure S1D). Moreover, the ATR inhibitor alone showed a bigger effect on CDC45 loading than the WEE1 inhibitor alone, while the WEE1 inhibitor showed bigger effects on CDK activity (Figure 1D). This finding is analogous to our previous result with CHK1 and WEE1 inhibitors [12]. We next investigated effects on cell survival. U2OS cells were treated with inhibitors alone or in combination for 24 h, and colony formation was assessed 12–14 days later. A clear synergistic reduction in clonogenic survival was observed after the combined treatment with 100 nM of each inhibitor (Figure 1E). We conclude that combined inhibition of WEE1 and ATR leads to a synergistic increase in S-phase DNA damage and reduction in clonogenic survival in U2OS cells. These results are largely similar to our previous findings obtained with combined inhibition of WEE1 and CHK1 [12].

### 2.2. Lung Cancer Cell Lines H460, A549, H1975, and SW900 Show Large Differences in Sensitivity to the WEE1 Inhibitor Despite a Similar Induction of CDK Activity

**Figure 2:**
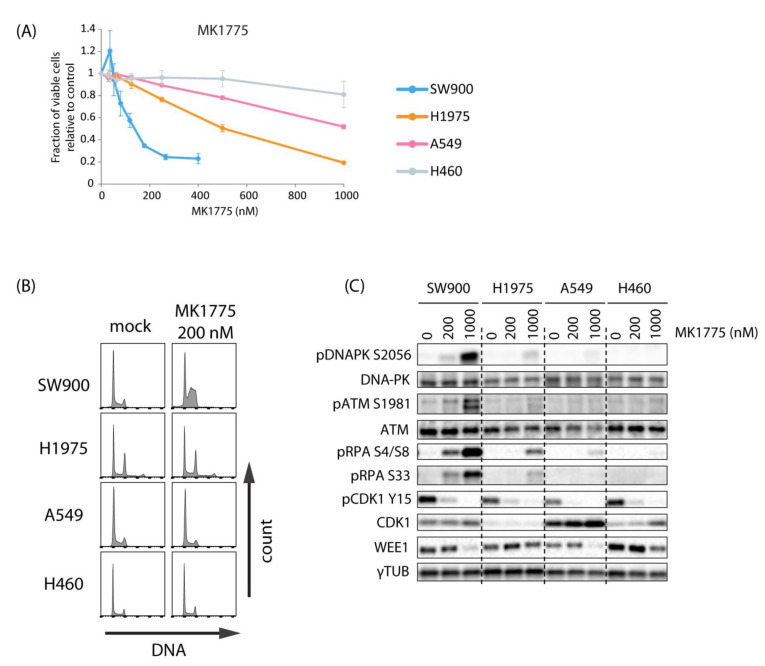
Lung cancer cell lines show large differences in sensitivity to the WEE1 inhibitor MK1775. (**A**) CellTiter-Glo viability assays of SW900, H1975, A549, and H460 cells treated with the indicated concentrations of MK1775 for 24 h and assayed at 5 days after drug removal (4 days for H1975). Error bars: SEM (*n* = 3). (**B**) Histograms showing cell cycle profiles (counts versus DNA content) from SW900, H1975, A540, and H460 cells treated with 200 nM MK1775 for 24 h. (**C**) Immunoblotting of extracts from H460, A549, H1975, and SW900 cells treated with 0, 200, and 1000 nM MK1775 for 24 h. The results in (**B**,**C**) are representative of two independent experiments.

To explore the potential of combined ATR and WEE1 inhibition for lung cancer treatment, we used a panel of four lung cancer cell lines with previously identified large differences in sensitivity to the WEE1 inhibitor MK1775 (sensitive SW900 > H1975 > A549 > H460 resistant) [26]. To better characterize the differences between these cell lines, we first addressed effects of the WEE1 inhibitor alone. Consistent with the previously published results, the four cell lines showed different sensitivities to MK1775, H460 being most resistant and SW900 most sensitive, as measured by CellTiter-Glo viability assays (Figure 2A and Table S1). Furthermore, MK1775-induced S-phase accumulation and phosphorylation of DNA damage markers ATM, DNA-PK, and RPA were highest in SW900 and lower in the more resistant cell lines (Figure 2B,C and Figure S4A), suggesting that induction of S-phase DNA damage parallels the loss of cell viability. Measurement of replication fork speed after treatment with the WEE1 inhibitor in the most resistant cell line H460 compared to U2OS cells confirmed that replication was unperturbed in H460 (Figure S4B–D). This is in agreement with high WEE1 inhibitor resistance and shows that the lack of S-phase accumulation in H460 was not due to the cells not cycling.

**Figure 3:**
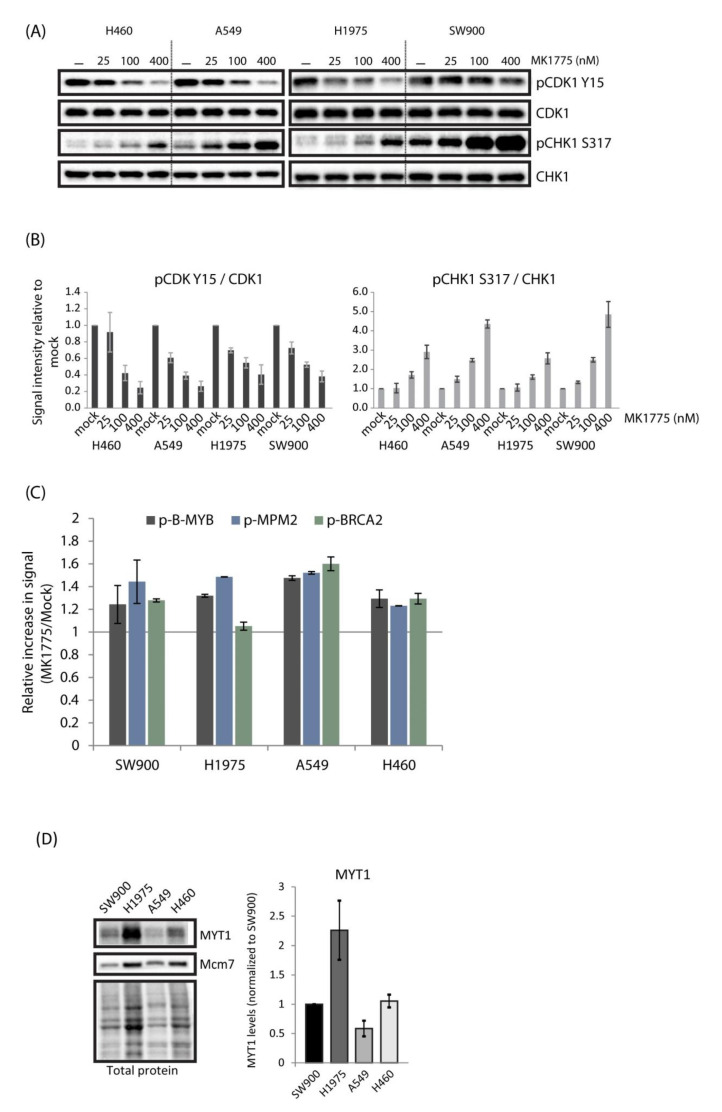
MK1775 increases CDK activity and CHK1 phosphorylation in lung cancer cell lines. (**A**) Immunoblotting of extracts from H460, A549, H1975, and SW900 cells treated with 0, 25, 100, and 400 nM MK1775 for 1 h. (**B**) Quantification of results from experiments as in A. Error bars: SEM (*n* = 3). (**C**) Flow cytometry analysis of phospho-B-MYB, phospho-MPM2, and phospho-BRCA2 in cells treated with 500 nM of MK1775 for 1 h, showing relative signal intensity (MK1775/mock) measured within a region of cells with S-phase DNA content, as indicated in Figure S3. (**D**) Immunoblotting of extracts from SW900, H1975, A549, and H460 showing MYT1 levels. Bar chart shows quantification of MYT1 levels relative to total protein and normalized to the value in SW900. Error bars: SEM (*n* = 3).

To address whether these differences might simply be a result of poor drug uptake in the most resistant cells, we examined, by immunoblotting, the phosphorylation of CDK1-Y15 and CHK1 S317 at 1h after treatment with a range of concentrations of MK1775 (25–400 nM). The results show that the inhibitor had about similar effects in all four lung cancer cell lines (Figure 3A,B). Furthermore, phosphorylation of the CDK targets B-MYB, MPM2, and BRCA2 was induced in all cell lines in response to WEE1 inhibition (Figure 3C and Figure S3). The levels of induction were comparable to that found in U2OS (Figure 1D), and even though slight differences were observed they did not correlate with MK1775 sensitivity (Figure 3C compared to Figure 2A). Thus, MK1775 appears to cause increased CDK activity and ATR activity in a similar manner in all the cell lines, excluding that variations in drug uptake could be a main reason for the differences in sensitivity. Interestingly, high levels of MYT1 kinase may limit MK1775-induced CDK activity, thereby causing resistance to WEE1 inhibition [27]. However, as A549 cells showed the lowest levels of MYT1 and were highly resistant to MK1775, MYT1 levels did not correlate well with WEE1 inhibitor resistance across our cell panel (Figure 3D). This further supports our finding that MK1775-induced CDK activity was similar in these cell lines.

### 2.3. Combined Inhibition of WEE1 and ATR Gives Variable Effects in the Lung Cancer Cell Lines

**Figure 4:**
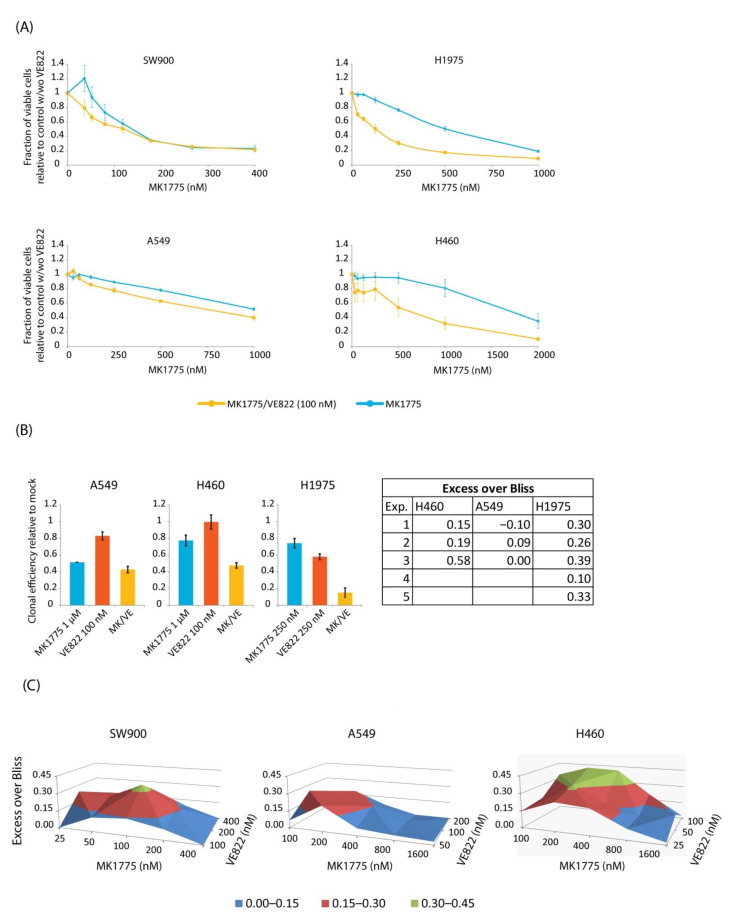
Cytotoxic effects of combined WEE1/ATR inhibition vary between lung cancer cells. (**A**) CellTiter-Glo viability assays of SW900, H1975, A549, and H460 cells from the same experiments as in Figure 2A, treated with MK1775 together with 100 nM VE822 (yellow) compared to the results of MK1775 alone (blue) shown in Figure 2A. Cell viability is normalized to mock for MK1775 alone and to VE822 alone for the combination. The fraction of viable cells after treatment with 100 nM VE822 alone was approximately 80% for A549 and H1975, 90% for SW900, and 60% for H460 cells. Error bars: SEM (*n* = 3). (**B**) Clonogenic survival assays of A549, H460, and H1975 cells treated with MK1775 and/or VE822 at the indicated concentrations for 24 h. Clonal efficiency relative to untreated cells is shown. Table to the right shows the difference between the measured effect and the expected additive effect (excess over Bliss) for the combined treatment in each experiment (*p* < 0.05 for H1975 cells). Error bars: SEM (*n* ≥ 3). (**C**) Excess over Bliss from CellTiter-Glo viability assays performed on SW900, A549, and H460 with a matrix of combinations of MK1775 and VE822. Cells were treated for 48h with inhibitors and assayed three days after drug removal.

Our finding that MK1775-induced increase in CDK activity was accompanied by phosphorylation of CHK1 S317 in all lung cancer cell lines suggests that ATR might have a protective role after WEE1 inhibition similar to that seen in U2OS. We therefore asked whether combined inhibition of ATR and WEE1 in the lung cancer cells would give a synergistic reduction in cell viability associated with induction of DNA damage in S-phase, such as was observed for U2OS cells (Figure 1). However, combined inhibition of WEE1 and ATR appeared to reduce cell viability to a different extent in the four lung cancer cell lines, showing more than additive effects in H460, H1975, and SW900, but little synergy in A549 (Figure 4A and Figure S5A and Table S1). We noted that in SW900 synergistic effects were only obtained for very low concentrations of MK1775 at which the drug had no or little effect alone. Moreover, addition of the ATR inhibitor could not fully compensate for cell resistance to the WEE1 inhibitor. (The cell lines being most resistant to the WEE1 inhibitor alone, H460 and A549, showed IC50 values for MK1775 of about 600–750 nM after the combined treatment compared to about 100 nM for H1975 and SW900). In these experiments the inhibitor treatment lasted 24 h and we applied a concentration of the ATR inhibitor (100 nM VE822) yielding 10–20% reduction in viability in all cell lines except in H460, which showed about 40% reduction in viability. Similar synergistic effects were obtained in H460 cells if the WEE1 inhibitor was combined with 50 nM VE822 (data not shown). To validate our findings, we performed colony formation assays with concentrations of the drugs expected to give synergistic effects. (A549, for which little synergy was observed, was treated with the same concentrations as H460). SW900 cells did not form colonies when cultured at low densities and could therefore not be used for this assay (unpublished observations). The effects on long-term survival were similar to those seen by the viability assay, with strong synergy observed only in H1975 and H460 cells, but not in A549 cells (Figure 4B). Of note, statistical analysis of Bliss scores indicate that the synergistic effects observed in H460 and SW900 were not significant (Figure S5A). However, the effect of the combined treatment in H460 was more than additive in all replicates of the experiments performed. Nonetheless, we wanted to more thoroughly explore the large synergistic effect in H460 and to test whether better synergy could be obtained in SW900 and A549 using other concentrations of the ATR inhibitor. To this end, we performed viability experiments with a matrix of concentrations of VE822 and the WEE1 inhibitor, with the inhibitor treatment lasting for 48 h. ATR inhibitor concentrations were chosen based on sensitivity to VE822 single treatment (Figure S5B). Combined treatment with MK1775 and VE822 gave a more than additive (excess over Bliss) reduction in viability in all cell lines (Figure 4C). However, the reduction in viability was significantly higher than the expected additive effect for more combinations in H460 than in the other two cells lines, and overall the synergy was also stronger (higher Bliss score) (Figure 4C and Figure S5C), thus confirming the results above (in Figure 4A,B). We concluded that combined treatment with WEE1 and ATR inhibitors gives a large synergistic reduction in cell viability in some, but not all, lung cancer cell lines.

**Figure 5:**
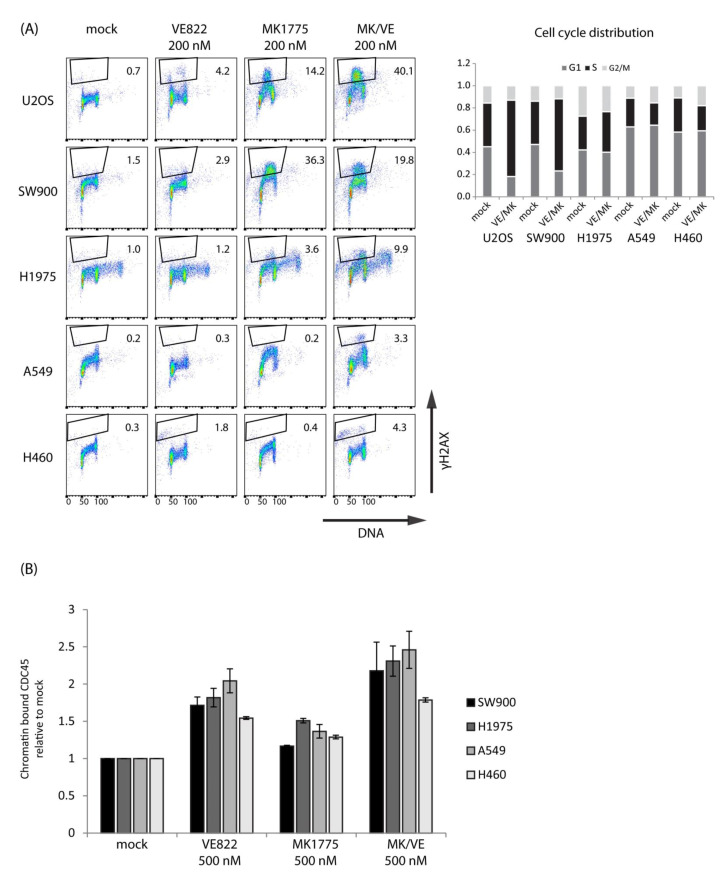
WEE1/ATR inhibition does not cause a large synergistic induction of γH2AX in lung cancer cells despite increased chromatin loading of CDC45. (**A**) Flow cytometry analysis of U2OS, SW900, H1975, A549, and H460 cells treated with MK1775 (200 nM) and/or VE822 (200 nM) for 24 h or left untreated (mock). Left: Scatter plots of γH2AX versus DNA content (Hoechst). Numbers indicate percentage of cells with strong γH2AX levels (within the marked regions). Results are representative of three similar independent experiments. Right: cell cycle analysis from mock and combination (MK/VE) samples showing the average fraction of cells in G1, S, and G2/M from three experiments. (**B**) Flow cytometry analysis of chromatin-bound CDC45 in lung cancer cell lines, treated as in Figure 1D and analyzed as in Figure S1D.

To investigate whether the reduction in cell viability in lung cancer cells after the combined WEE1/ATR inhibitor treatment was associated with increased S-phase DNA damage, we performed flow cytometry analysis of γH2AX induction. First, we wanted to compare the effect to that observed in U2OS and treated cells for 24 h with 200 nM of MK1775 and VE822 alone and in combination. In U2OS cells the combined treatment with MK1775 and VE822 gave a large synergistic increase of DNA damage in S-phase, but no or only small increases were observed in the lung cancer cell lines (Figure 5A). Furthermore, an accumulation of cells in S-phase was only observed in U2OS and SW900 upon combined treatment, however, in SW900 the effect was only marginally larger than that seen for single treatment with MK1775 (compare Figure 5A to Figure S4A). To rule out the possibility that combination treatment with a higher concentration of MK1775 would give a large synergistic induction of DNA damage in the most resistant lung cancer cell lines, we repeated the flow cytometry analysis of γH2AX in H460, A549, and H1975 cells treated with a dose of MK1775 that gave ~50% loss in viability and combined it with 100 nM of VE822. Again, a large synergistic effect was not obtained (Figure S2A). In both experiments above, we also examined the drug’s effect on mitotic entry by staining for phospho-H3 but found no large synergistic increase in mitotic fraction that could explain the synergy observed in the viability experiments (Figure S2B,C). To investigate potential mechanisms behind why lung cancer cell lines lacked a synergistic induction of S-phase DNA damage, we asked whether drug-induced loading of CDC45 might be defective. However, as seen in U2OS (Figure 1D), CDC45 loading at 1 h after treatment with 500 nM of both MK1775 and VE822 was increased in all cell lines and was larger than for treatment with each inhibitor alone (Figure 5B). This suggests that the combined treatment similarly increases replication initiation in U2OS and lung cancer cell lines, although to a slightly lesser extent in H460 than in the other cell lines. Together, these results suggest that despite elevated replication initiation, the reduction in viability upon combined treatment with WEE1 and ATR inhibitors cannot simply be explained by an increased induction of S-phase DNA damage in the lung cancer cells.

### 2.4. Less Synergy Is Observed between WEE1/ATR Inhibitors When Cells Are Co-Treated with Radiation

**Figure 6:**
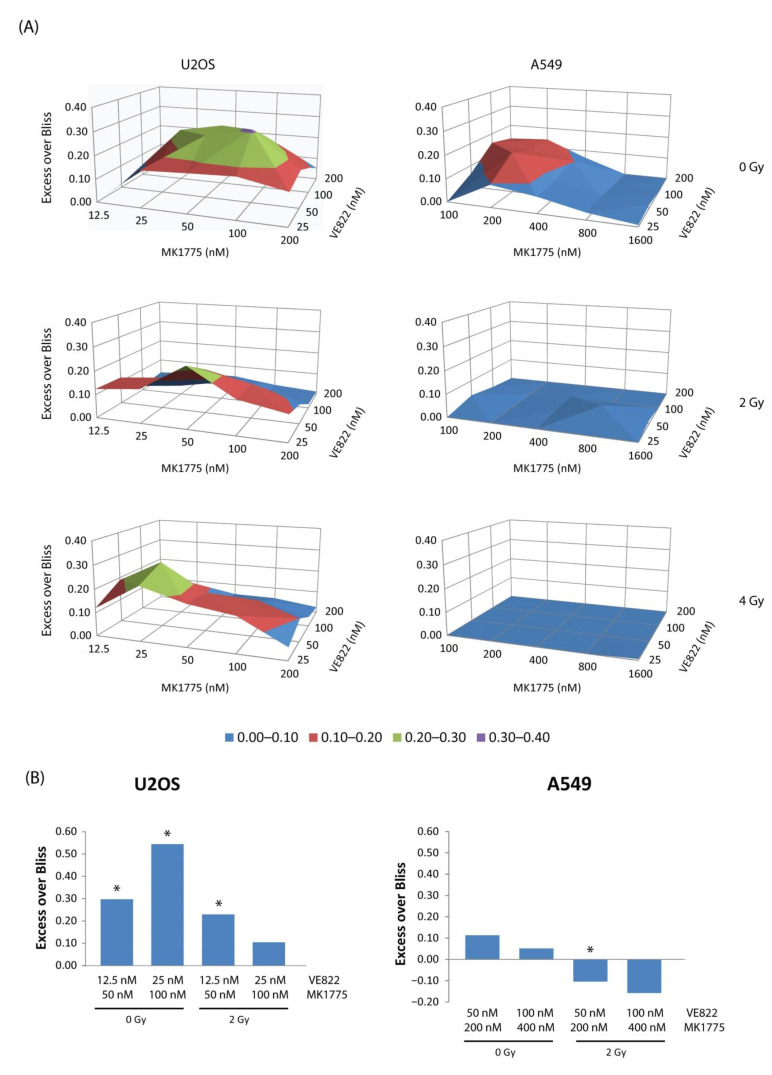
Synergy between MK1775 and VE822 is weakened when combined with radiation. (**A**) U2OS and A549 cells were seeded in 96-well plates pre-printed with a matrix of different concentrations of VE822 and MK1775 alone and in combination and exposed to X-ray radiation at doses of 2 and 4 Gy or left unexposed. Drugs were washed out 48 h after treatment and viability measured 3 days after drug removal using CellTiter-Glo assays. Average synergy scores (excess over Bliss) for each dose pair are presented in surface plots (*n* = 4). The average fraction of viable cells after 2 and 4 Gy were 0.4 and 0.2 (U2OS) and 0.6 and 0.4 (A549). (Data for 0 Gy in A549 are from the same experiment as in Figure 4C). (**B**) Clonogenic assays of U2OS and A549 treated with two different concentrations of VE822 and MK1775 alone and in combination and exposed to X-ray radiation at a dose of 2 Gy or left unexposed. Cells were left with drug on for 24 h and incubated further without drug until colonies were formed (12–14 days). Average synergy score (excess over Bliss) for combined treatment is plotted in bar graphs (*n* = 3). The average fraction of viable cells after 2 Gy were 0.4 (U2OS) and 0.6 (A549). *p* values were determined by the two-tailed Student’s one-sample *t* test, * *p* < 0.05.

Both WEE1 and ATR inhibitors have been shown to radiosensitize cancer cells [28,29,30,31,32,33,34], and are in clinical testing together with ionizing radiation (IR). Radiosensitization is thought to occur through cell cycle checkpoint abrogation, inhibition of DNA repair, and induction of DNA damage in S-phase [32]. To better evaluate the clinical potential of combined WEE1/ATR inhibition, we therefore addressed whether the observed synergy was maintained or possibly enhanced in the presence of additional radiation treatment. Furthermore, we wanted to compare a cell line that showed large synergistic effects to one showing weak synergy. Thus, to explore this, we performed cell viability assays of U2OS and A549 cells treated with X-ray radiation (2 and 4 Gy) and a matrix of different concentrations of MK1775 and VE822 alone and in combination. Since both checkpoint induction and DNA damage repair occurs within the first 48 h after irradiation, we added the inhibitors before irradiation, kept them in the cell medium for 48 h, and measured viability via CellTiter-Glo assay 5 days after irradiation. As expected, the combined treatment gave stronger synergy in U2OS than in A549 in non-irradiated cells (Figure 6A, top panel). Strikingly, the synergy was overall weakened in both cell lines upon exposure to radiation (Figure 6A, mid and bottom panels). In U2OS cells there was still some synergy detected in the irradiated samples, but interestingly the inhibitor concentrations that gave synergy were lower compared to that seen in the 0 Gy samples (Figure 6A, compare areas of green color in the U2OS plots). Cell cycle analysis of U2OS showed a transient accumulation in S- and G2-phase after IR as expected due to checkpoint activation and as measured at 6 h after treatment (Figure S6B, left). Of note, no further increase in the S-phase population was observed in inhibitor-treated irradiated cells at this time point, which might help explain why effects are different in irradiated versus non-irradiated cells. On the other hand, the highest inhibitor concentrations caused a strong S-phase accumulation regardless of IR at 24 h after treatment (Figure S6B, second left chart). In A549, no accumulation of cells in S-phase was observed in irradiated cells at 6 h (likely because of a stronger G1 checkpoint in this cell line), and after 24 h the irradiated samples with and without inhibitors had very few cells in S-phase (Figure S6B, right). The cell cycle effects after combined inhibitor and IR treatment are complex, likely because the inhibitors cause checkpoint abrogation and inhibition of DNA repair in addition to inducing S-phase damage. To explore whether the above findings on cell viability were reflected when studying long-term survival, we performed clonogenic assays of U2OS and A549 cells treated with two concentrations of MK1775 and VE822 alone and in combination and compared non-irradiated cells to cells exposed to X-rays at a dose of 2 Gy. The results were similar to those obtained in the viability assays, showing larger synergistic effects in U2OS than in A549 both in the absence and presence of IR, and a weakened synergy, or even antagonism (A549), in cells exposed to X-rays as compared to non-irradiated cells (Figure 6B). Of note, the ATR inhibitor alone showed a bigger radiosensitizing effect than the WEE1 inhibitor alone in both cell lines, with the highest effect in U2OS (Figure S6A,C). Bliss independence analysis of synergy between IR and VE822, MK1775, or combined VE822/MK1775 also showed the overall biggest effects for the ATR inhibitor alone (Figure S6D). In these cell lines, the ATR inhibitor thus appears most promising to use for radiosensitization. Altogether, these results suggest that no major benefit can be obtained from combining dual WEE1/ATR inhibition with radiotherapy, but again the magnitude of the effects varies between cell lines.

### 2.5. No Correlation Is Found between Expression Levels of Biomarkers Associated with WEE1 and ATR Inhibitor Sensitivity and Observed Differences in Sensitivity in Lung Cancer and U2OS Cells

**Figure 7:**
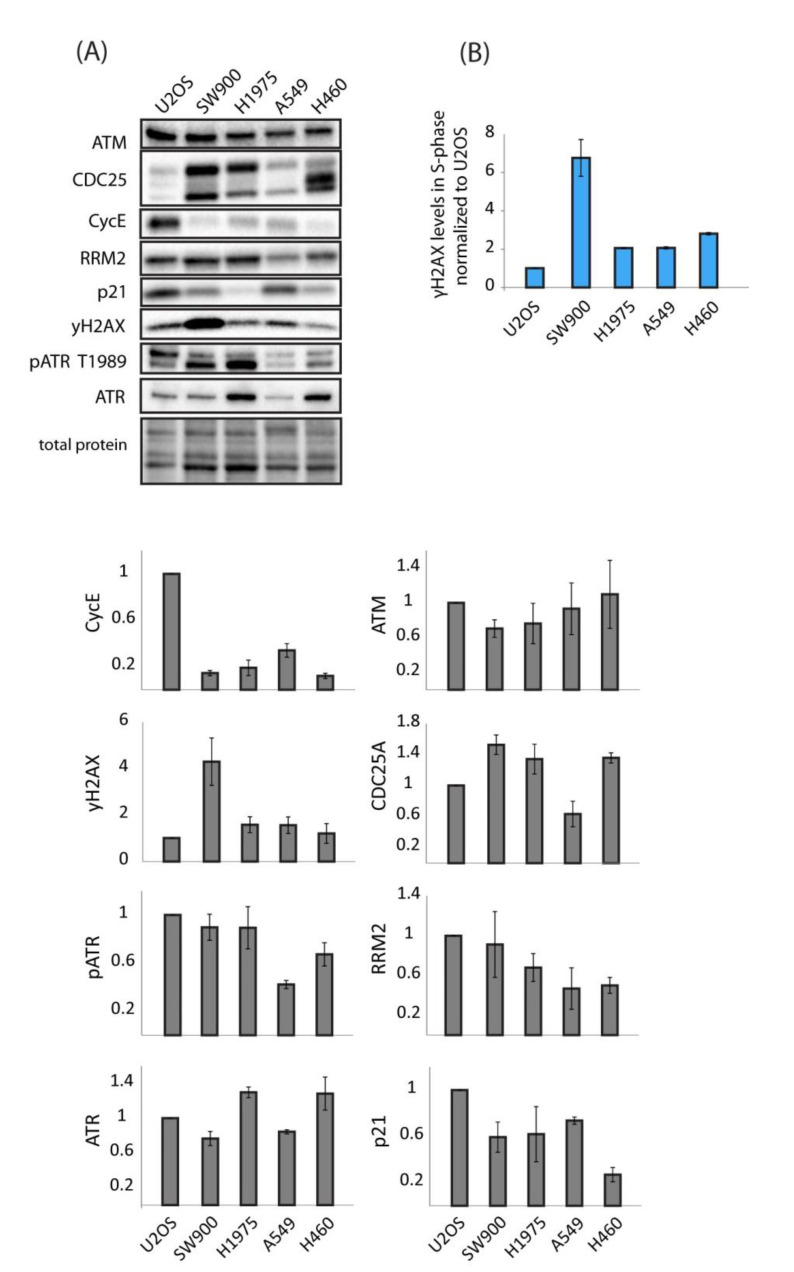
Expression levels of proposed biomarkers of WEE1 and ATR inhibitor sensitivity in lung cancer and U2OS cell lines. (**A**) Immunoblot of lysates from exponentially growing cells. Bar charts (grey) show quantifications of protein levels relative to total protein and normalized to the levels in U2OS. Error bars: SEM (*n* ≥ 3). (**B**) Flow cytometry analysis of γH2AX levels in S-phase of exponentially growing cells normalized to U2OS. Error bars: SEM (*n* = 2).

The panel of cell lines tested in our experiments showed widely different sensitivities and responses to treatment with ATR and WEE1 inhibitors both alone and in combination. A number of sensitivity/resistance markers have been proposed for WEE1 and ATR inhibitors. We set out to test whether some of these markers could account for the differences in sensitivity observed in our cell lines. First, it should be pointed out that no positive correlation between WEE1 inhibitor sensitivity and ATR inhibitor sensitivity was observed in our lung cancer cell lines (compare Figure 2A to Figure S5B). The protein expression levels of Cyclin E, cdc25A, ATM, p21, RRM2, γH2AX, and ATR/pATR were assessed via immunoblotting on samples from exponentially growing, untreated U2OS and lung cancer cells (Figure 7A). In addition, we measured S-phase levels of γH2AX by flow cytometry (Figure 7B). We found no clear correlation between WEE1 or ATR inhibitor sensitivity and the markers investigated. For instance, H460 and U2OS were most sensitive to the ATR inhibitor (Figures S5B and S6A), but none of the markers were outstanding for these two cell lines (Figure 7A,B). Furthermore, U2OS and SW900 were most sensitive to the WEE1 inhibitor (Figure 2A and Figure S6A), and these two cell lines did not have a common marker (Figure 7A,B). However, cyclin E levels were comparatively very high in U2OS cells and γH2AX levels very high in SW900 cells. In addition, they both had relatively high levels of phosphorylated ATR. Together, this could indicate elevated endogenous replication stress in these cell lines, which might be consistent with their high sensitivity to the WEE1 inhibitor. On the other hand, high γH2AX levels would be expected also in U2OS if they were suffering from replication stress. Finally, U2OS, H1975, and H460 showed the biggest synergist effects upon the combined treatment (Figure 1, Figure 4 and Figure 6A), but no marker showed a distinct expression pattern in these cell lines. We concluded that no single biomarker, among the ones tested, could likely account for the differences in sensitivity observed in our panel of cell lines exposed to WEE1 and/or ATR inhibitors.

## 3. Discussion

Due to their protective roles in the replication stress response and in G2 checkpoint control, ATR and WEE1 are attractive therapeutic targets. Cancer cells often show elevated replication stress due to, for example, oncogene expression or tumor hypoxia [35,36], which likely provides sensitivity to ATR or WEE1 inhibitors. Furthermore, loss of the G1 checkpoint is frequent in tumors [37] and potentially provides increased reliance on the G2 checkpoint, thereby selectively sensitizing cancer cells to checkpoint inhibitors [4,34,38]. The ATR downstream factor CHK1 shows similar roles in replication stress and the G2 checkpoint as ATR, but ATR inhibitors may be better suited for the clinic since CHK1 inhibitors have shown problems with normal tissue toxicity [39]. The approach of simultaneous inhibition of WEE1 and ATR has emerged very recently and appears promising based on preclinical studies of breast cancer and AML [19,20,21]. To further evaluate this new exciting approach, we investigated the effects of combined ATR/WEE1 inhibition in U2OS osteosarcoma and four lung cancer cell lines. Our study shows, for the first time, effects of this combination in lung cancer. Furthermore, as opposed to focusing only on the most sensitive cell lines, we particularly included cell lines that are resistant to WEE1 inhibition. Moreover, we explored the effects of simultaneous ATR/WEE1 inhibition together with radiation, which is important since radiotherapy is a standard treatment highly relevant to combine with such inhibitors.

The combined treatment with ATR and WEE1 inhibitors caused a large synergistic reduction in cell viability in U2OS, H460, and H1975 cells, while the effects were weaker in SW900 and close to additive in A549 cells. Furthermore, a large synergistic induction of DNA damage and accumulation of cells in S-phase accompanied the reduction in cell viability upon the combined treatment in U2OS cells, but not in H1975 or H460 cells. These results demonstrate that combined ATR/WEE1 inhibition can cause very different effects in different cancer cell lines. These differences are likely related to the fact that both of these inhibitors cause multiple cellular effects, affecting, for example, DNA replication, homologous recombination repair, G2 checkpoint control, as well as mitotic events [2,3,5,10,40,41,42,43,44]. It is possible that S-phase DNA damage is highly important in U2OS cells upon treatment with the inhibitors, while the other effects, or specific combinations of them, may be more important in other cell lines. Notably, such differences may have huge implications for biomarker development. Biomarkers are highly needed for prediction of responses to these inhibitors in cancer patients and several biomarkers have been suggested, such as p53 mutation, replication stress associated markers (γH2AX, oncogene expression and low levels of ribonucleotide reductase subunit RRM2) [3,22,45,46], and multiple other regulators of DNA damage and cell cycle, such as ATM, CDC25A, MYT1, p21, or ATR signaling [27,47,48,49,50]. We have analyzed and compared protein levels across our cell lines of several of these markers but have not been able to find one unifying mechanism that can explain all the different effects observed. Oncogene-associated cyclin E levels were comparatively high in U2OS cells, indicating elevated endogenous replication stress, but did not correlate with high expression of the replication stress marker γH2AX. By contrast, SW900 showed high levels of γH2AX in line with WEE1 inhibitor sensitivity, but on the other hand it was not particularly sensitive to ATR inhibition, also for which γH2AX is a proposed sensitivity marker. Furthermore, even though p53 mutation status correlated with WEE1 inhibitor sensitivity in our panel of lung cancer cells (the two most resistant cell lines, H460 and A549, have wt-p53 and the two most sensitive cell lines, H1975 and SW900, have mut-p53), it did not correlate with sensitivity to ATR inhibition (H460 is most sensitive). Moreover, U2OS, which was highly sensitive to WEE1 inhibition, also has wt-p53, indicating that no strict correlation between p53 status and sensitivity to WEE1 inhibition exists. This is further corroborated by a previous study where a large panel of lung cancer cell lines was examined [26]. We concluded that WEE1 and ATR inhibitors affect multiple mechanisms in the cells, and it might therefore be difficult to find one universal biomarker for the treatment response to these inhibitors. More likely, a panel of several biomarkers will be useful.

Interestingly, after WEE1 inhibition alone, the cell lines included in our study showed similar induction of CDK activity despite very different reductions in cell viability. This could indicate that the resistant cell lines better tolerate high CDK activity during DNA replication, avoiding deleterious S-phase DNA damage. High CDK activity after WEE1 inhibition can cause unscheduled replication initiation, replication stalling, and activation of endonucleases such as MUS81 in S-phase [5,10,11]. One might speculate that MK1775-induced activation of MUS81 endonuclease is defective in the resistant cell lines. In line with this, we observed that MUS81 chromatin binding assessed via flow cytometry was increased in U2OS but not in H460 cells upon treatment with MK1775 (Figure S7). This result may indicate that activation of MUS81 is defective in H460 cells, or that less substrate for MUS81 is available after WEE1 inhibition in H460 compared to U2OS. Another possibility is that the most resistant cell lines, H460 and A549, better tolerate replication stress due to mechanisms related to, for example, translesion synthesis. A recent study showed that cancer cells can display different sensitivities to an inhibitor of translesion synthesis, suggesting that this type of replication is more important in some cancer cell lines than others [51]. Interestingly, A549 cells were sensitive and U2OS cells very resistant to this inhibitor [51], indicating that A549 uses more translesion synthesis than U2OS. This could potentially be an underlying reason why A549 better tolerates MK1775- induced CDK activity than U2OS cells.

We noted that the ATR inhibitor alone showed a bigger effect on CDC45 loading than the WEE1 inhibitor alone, while the WEE1 inhibitor showed a bigger effect on CDK activity (Figure 1D and Figure 5B). While WEE1 mainly regulates CDC45 loading due to its role in suppressing CDK activity, ATR also appears to regulate CDC45 loading in a manner independent of CDK, similar to our previous finding for CHK1 [12]. However, although CDC45 loading was higher, the ATR inhibitor induced less phosphorylation of DNA damage markers and less accumulation of cells in S-phase than the WEE1 inhibitor, consistent with less S-phase DNA damage (Figure 1A–C and Figure 5A). The largest increase in CDC45 loading was seen for the combined treatment, but even if this increase was similar in all cell lines very little DNA damage induction was observed in three of the lung cancer cell lines. Thus, in contrast to our previous results with CHK1/WEE1 inhibition [12], there is no strict correlation between CDC45 loading and induction of S-phase DNA damage when considering ATR/WEE1 inhibition.

Our measurements of cell viability in irradiated cells showed less synergy between ATR and WEE1 inhibition compared to the effect seen in the absence of IR. We also noted that ATR inhibition had a bigger radiosensitizing effect than WEE1 inhibition (Figure S6). Together, these measurements in U2OS and A549 cells suggest that simultaneous inhibition of WEE1 and ATR may not be particularly advantageous in combination with radiotherapy. However, we cannot exclude that other advantageous effects not studied here might occur in vivo. Interestingly, both WEE1 and ATR inhibitors were shown to affect anti-tumor immune responses after radiation [52,53,54,55], and simultaneous ATR/WEE1 inhibition together with radiotherapy could potentially be more beneficial in this aspect.

## 4. Materials and Methods

### 4.1. Cell Culture and Drug Treatments

Human NCI-H460 and A549 lung cancer (ATCC) and U2OS osteosarcoma cells (all p53 wt) were cultured in Dulbecco’s modified Eagle’s medium (DMEM), and SW900 and H1975 lung cancer cells (p53 mutated) in Roswell Park Memorial Institute (RPMI) medium (both media from Gibco, Grand Island, NE, USA), at 37 °C in a humidified atmosphere with 5% CO_2_. The media were supplemented with 10% fetal bovine serum (Biowest, Nuaillé, France) and 1% penicillin/streptomycin (Gibco, Grand Island, NE, USA). All cell lines were verified via short tandem repeat (STR) technology, as described previously [12]. The WEE1 inhibitor MK1775 (AZD1775) was from Merck Calbiochem. The ATR inhibitors VE822 and AZD6738 were from Selleck Chemicals.

### 4.2. Flow Cytometry Analysis of CDK Targets, Chromatin-Bound CDC45 and MUS81, DNA Damage, and Cell Cycle Distribution

For analysis of protein phosphorylation, cells were fixed with 70% ethanol and stained with antibodies, as described previously [12]. The primary antibodies were mouse anti-phospho-γH2AX(S139) (05-636, Millipore, Darmstadt, Germany), rabbit anti-phospho-H3(S10) (06-570, Millipore), and three antibodies to CDK targets: rabbit anti-phospho-B-MYB(T487) (ab76009, Abcam, Cambridge, UK), rabbit anti-phospho-BRCA2(S3291) (AB9986, Millipore), and mouse anti-phospho-S/T-P MPM-2 (05-368, Millipore). Secondary antibodies were Alexa Fluor 488 and 647 (Molecular Probes, Eugene, OR, USA) and Cy3 (Jackson ImmunoResearch, Cambridgeshire, UK) anti-mouse and anti-rabbit IgG. For analysis of chromatin-bound proteins, cells were pre-extracted and fixed, as described previously [12], but with the following modifications for CDC45 in SW900 and H1975 and MUS81: cell pellets were treated with 100 µL extraction buffer (20 mM HEPES, pH 7.9; 1.5 mM MgCl_2_; 50–140 mM NaCl; 300 mM sucrose; and 0.5% Triton TX-100) for 5 min on ice. The cells were then fixed by adding 900 µL of 10% formalin solution (HT501128 SIGMA) and undergoing incubation for 10 min at room temperature. Cells were stained with anti-CDC45 (sc-55569, Santa Cruz) or anti-MUS81 (ab14387, Abcam), followed by Alexa Fluor 488 anti-mouse IgG. In experiments where median values were measured, barcoding of sets of up to four samples with Pacific Blue was used as before [12] or as described in Figure S3 to eliminate variation in antibody staining between the individual samples. The DNA stain FxCycle^TM^ Far Red (200 nM FxCycle and 0.1 mg/mL RNase A) (Thermo Fisher Scientific) was used in barcoding experiments and Hoechst 33258 (1.5 µg/mL) in other experiments. Median values were measured within a region corresponding to cells with an S-phase DNA content. Flow cytometry analyses were performed on an LSRII flow cytometer (BD Biosciences, Franklin Lakes, USA) using Diva and FlowJo software. For cell cycle analysis the built-in Watson Pragmatic algorithm in FlowJo was used.

### 4.3. Viability Assay (CellTiter-Glo)

Cells were seeded in 96-well microplates (Nunc™, VWR) at a density of 500 (H460, A549, U2OS), 800 (H1975), or 1000 (SW900) cells/well. Six to twenty-four hours after seeding, cells were treated with MK1775 in a seven-step gradient (36, 52, 80, 118, 178, 266, and 400 nM for SW900 and 31.25, 62.5, 125, 250, 500, and 1000 nM for H460, A549, and H1975 (plus 2000 nM for H460) alone or in combination with 100 nM of ATR inhibitor VE822. After 24 h, inhibitors were removed, cells washed once with PBS, and fresh medium without inhibitors was added. Four to five days after inhibitor removal, the CellTiter-Glo assay (Promega, Madison, WI, USA) was used to measure metabolic activity as a readout for viable cells. Of note, we previously compared measurements at 4, 5, and 6 days after drug removal in analogous experiments and obtained similar results at all three days [56]. In short, an equal volume to culture volume of diluted CellTiter-Glo reagent was added to each well, plates incubated while shaking for 10 min at room temperature, and luminescence read in a Tecan Spark multimode microplate reader with integration time set to 1 sec. Average values from triplicate wells were obtained in three independent experiments. For experiments done with a matrix of different concentrations of WEE1 and ATR inhibitors with or without combined radiation treatment, cells were seeded at densities of 500/1000/1500 cells/well (U2OS 0 Gy/2 Gy/4 Gy), 800/1200/1800 cells/well (A549 0 Gy/2 Gy/4 Gy), 800 cells/well (H460), and 1000 cells/well (H1975 and SW900) in plates pre-printed with drugs. Immediately after seeding, cells were irradiated in a Faxitron X-ray machine (160 kV, 6.3 mA, 1 Gy/min) at doses of 2 and 4 Gy or left unexposed. Drugs were washed out 48 h after treatment and viability measured 3 days after drug removal (5 days after irradiation). This time point was selected based on a previous study showing a robust correlation between the radiosensitizing effects of drugs measured using clonogenic assays and cell growth/viability assays performed at 4–6 days after irradiation [57]. Average values from duplicate wells were obtained in four independent experiments. Synergy was determined via Bliss independence using the fraction affected (Fa) of treated cells relative to no-drug samples. The expected effect of the combination will then be: (Fa_(MK1775)_ + Fa_(VE822)_ − Fa_(MK1775)_ × Fa_(VE822)_). Excess over Bliss, calculated as the difference between the measured effect and the expected effect, was plotted on surface charts using Excel. Statistical significance (*p* < 0.05) was determined via one-sample two-tailed Student *t* test (test criterion: excess over Bliss ≠ 0).

### 4.4. Clonogenic Survival Assays

One hundred fifty to three hundred cells were seeded in 6 cm culture dishes (BD Biosciences) in triplicate with medium containing various concentrations of WEE1 and/or ATR inhibitors (MK1775 and VE822). After 24 h, the medium was replaced by 4 mL fresh medium without inhibitors. Cells were then cultured for an additional 12-14 days, fixed in 70% ethanol, and stained with methylene blue. Colonies of 50 or more cells were counted as survivors. For assays in which inhibitor treatment was combined with radiation exposure, 150–300 cells (0 Gy) and 450–2000 cells (2Gy) were seeded 24 h prior to treatment. Cells were irradiated immediately after adding inhibitors, then cultured for 24 h with inhibitors and processed further, as described above. Survival fractions were calculated in each experiment as the average cloning efficiency (from 3 parallel dishes) after treatment with the inhibitors divided by the average cloning efficiency for non-treated cells.

### 4.5. Immunoblotting

Cells were lysed in ice-cold TX-100 buffer (100 mM NaCl, 50 mM Tris pH 7.5, 2 mM MgCl_2_, 0.5% TX-100) containing 100 U/mL Benzonase (Sigma-Aldrich, St. Louis, MO, USA). After 1 h incubation on ice, Lane Marker Reducing Sample Buffer (Pierce Biotechnologies, Waltham, MA, USA) was added and samples were boiled (95 °C, 5 min). Criterion TGX gels (BioRad, Hercules, CA, USA) and nitrocellulose membranes (BioRad) were used for separation and transfer, respectively. The following antibodies were used for blotting: mouse anti-CDK1 (9112, Cell Signaling, Danvers, MA, USA), rabbit anti-phospho-CDK1(Y15) (9111, Cell Signaling), mouse anti-γ-Tubulin (T6557, Sigma-Aldrich), mouse anti-DNA-PK (MA5-13238, Thermo Fisher Scientific, Waltham, MA, USA) rabbit anti-phospho-DNA-PK (S2056) (ab18192, Abcam), mouse anti-ATM (clone AM9, Upstate), mouse anti-phospho-ATM (S1981) (4526, Cell Signaling), rabbit anti-ATR (2790, Cell Signaling), rabbit anti-phospho-ATR (T1989) (GTX128145, GeneTex, Irvine, CA, USA), rabbit anti-phospho-RPA (S4/S8) (A300-245, Nordic Biosite, Täby, Sweden), rabbit anti-phospho-RPA (S33) (A300-246A, Nordic Biosite), mouse anti-Cdc25A (DCS-120) (sc-56264, Santa Cruz, Dallas, TX, USA), rabbit anti-phospho-CHK1 (S317) (2344, Cell Signaling), mouse anti-CHK1 (DCS310.1, Santa Cruz), mouse anti-MCM7 (sc-65469, Santa Cruz), mouse anti-phospho-γH2AX(S139) (05-636, Millipore), rabbit anti-MYT1 (4282, Cell Signaling), rabbit anti-CyclinE (C-19, sc-198, Santa Cruz), goat anti-RRM2 (sc-10846, Santa Cruz), rabbit anti-p21 (H-164, SC-756, Santa Cruz), and rabbit anti-WEE1 (4936, Cell Signaling). Peroxidase-conjugated secondary antibodies were from Jackson Immunoresearch and SuperSignal HRP chemoluminescent substrate from Thermo Fisher Scientific was used for detection. In Figure 1A, Figure 2C and Figure S1B samples were run on multiple gels and a representative blot of loading controls; MCM7 and γ-Tubulin are shown.

### 4.6. siRNA Transfection

U2OS cells were transfected using RNAimax (Thermo Fisher), according to the manufacturer’s protocol. Oligonucleotides used were: Wee1-1, GGAAAAAGGGAAUUUGAUG (Sigma-Aldrich) and Scr, GGUUUCUGUCAAAUGCAAACGGCUU (Thermo Fisher).

### 4.7. DNA Fiber Assay

The DNA fiber assay was performed similarly as we did in a previous study [58]. Briefly, U2OS and H460 cells were pulse labelled with 25 μM CldU, followed by 250 μM IdU for 20 min each. After labelling, cells were harvested in ice-cold PBS. DNA fiber spreads were prepared by spotting 2 μL of cells (1 × 10^5^ cells/mL in PBS) onto microscope slides, followed by lysis with 7 μL of spreading buffer (0.5% SDS, 200 mM Tris-HCl pH 7.4, and 50 mM EDTA) for 5 min. For spreading, slides were tilted and further fixated in methanol/acetic acid (3:1). Prior to immunodetection, slides were treated with 2.5 M HCl for 1 h and 15 min. The slides were further stained for 1 h with rat anti-bromodeoxyuridine (Clone BU1/75, Abcam) to detect CldU and mouse anti-bromodeoxyuridine (Clone B44, BD Biosciences) to detect IdU. Subsequently, slides were fixed in formalin solution for 10 min and further incubated with anti-rat IgG Alexa Fluor 568 and anti-mouse IgG Alexa Fluor 488 (Molecular Probes) for 2 h. Slides were mounted with Fluoroshield. Images were acquired with an AxioImager Z1 ApoTome microscope system (Carl Zeiss, Jena, Germany) using a 63× (1.4 numerical aperture) oil lens equipped with an AxioCam Mrm camera and the Axiovision 4.8.2 (Carl Zeiss) software. Images were analyzed using ImageJ (https://imagej.nih.gov/ij/index.html, accessed on 1 February 2019), where at least 200 fibers were measured per condition in each experiment. Replication track lengths were calculated using the conversion factor 1 μM = 2.59 kb. Replication structures were quantified using the multi-point tool.

## 5. Conclusions

In conclusion, we investigated the effects of WEE1 and ATR inhibitors, alone and in combination, on U2OS osteosarcoma cells and on a panel of four lung cancer cell lines with different sensitivities to the WEE1 inhibitor. WEE1 was equally well-inhibited in all cell lines, indicating that resistance to WEE1 inhibitory treatment is not a result of poor efficacy of the inhibitor in general but is likely due to an effect downstream of CDK activation. We observed large synergistic effects in some, but not all, of these cell lines when combining ATR and WEE1 inhibitors, and the mechanism(s) of synergy seemed to vary between cells lines. Moreover, the combined treatment could not fully compensate for cell resistance to the WEE1 inhibitor. Thus, one should critically consider which patients would potentially benefit from combined ATR/WEE1 inhibition, being careful not to generalize based on individual biomarkers and to take into account sensitivity to single treatments.

## Data Availability

The data presented in this study are available on request from the corresponding author.

## Supplementary Materials

The following are available online at https://www.mdpi.com/article/10.3390/cancers13153790/s1, Figure S1: WEE1 and ATR dependency of synergy is verified by a second ATR inhibitor and siRNA-mediated depletion of WEE1, Figure S2: In lung cancer cell lines synergistic reduction in survival does not correlate with DNA damage induction in S-phase or with premature mitosis, Figure S3: CDK activity measurements in lung cancer cell lines, Figure S4: Resistant cell lines show no accumulation of cells in S-phase and continue replication in the presence of WEE1 inhibitor, Figure S5: Synergy scores and ATR inhibitor sensitivity in lung cancer cell lines, Figure S6: Radiosensitizing effect of WEE1 and ATR inhibitors in U2OS and A549, Figure S7: Chromatin loading of MUS81 is more increased in U2OS than in H460 in response to WEE1 inhibition, Table S1: Approximate IC50 values (MK1775 and VE822) in lung cancer cell lines based on Cell-Titer-Glo experiments.
