# Supplementary material for: Differential Effects of Combined ATR/WEE1 Inhibition in Cancer Cells
Source: Cancers (Basel). 2021 Jul 28;13(15):3790. doi: 10.3390/cancers13153790 (PMC8345075; doi:10.3390/cancers13153790)
Supplement: Supplementary file 1 [file cancers-13-03790-s001.zip › cancers-1306089-SI.pdf]

Supplementary Materials: Differential Effects of Combined ATR/WEE1 Inhibition in Cancer Cells

Gro Elise Rødland, Sissel Hauge, Grete Hasvold, Lilli T. E. Bay, Tine T. H. Raabe, Mrinal Joel and Randi G. Syljuåsen

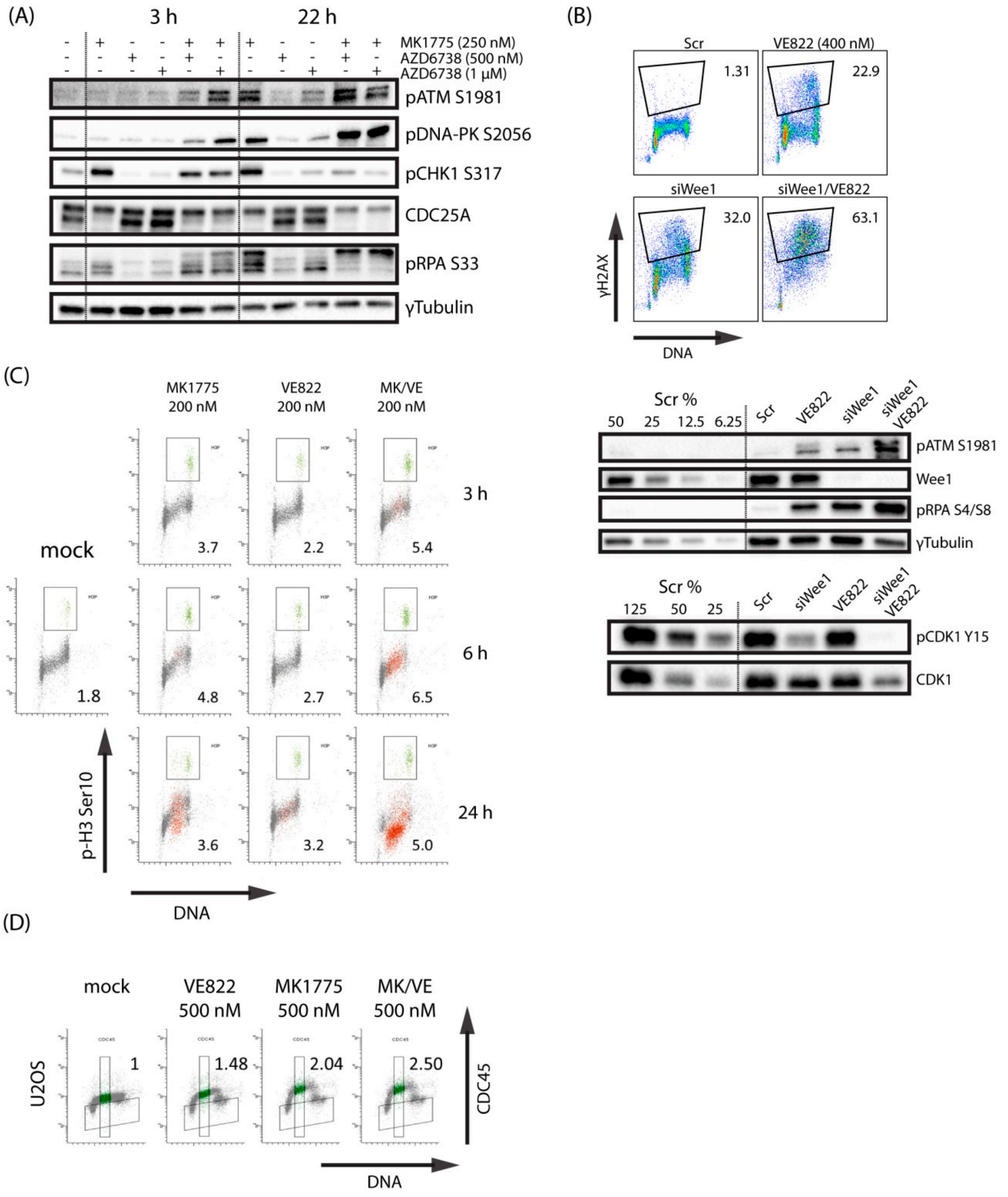

Figure S1. WEE1 and ATR dependence of synergy is verified by a second ATR inhibitor and siRNA depletion of WEE1. **(a)** Immunoblotting of U2OS cells treated with the ATR inhibitor AZD6738 (500 nM or 1 μM) and/or WEE1 inhibitor MK1775 (250 nM) for 3 h or 22 h. **(b)** Flow cytometry analysis of U2OS cells transfected with WEE1 siRNA and treated with VE822 (400 nM) for 24 h, as indicated. Plots of γH2AX versus DNA content (Hoechst) are shown. Numbers indicate % cells with strong γH2AX levels. WEE1 knock down efficiency is shown on immunoblot, together with DNA-damage markers and pCDK1. **(c)** Flow cytometry analysis of phospho-H3 versus DNA content (Hoechst) from the same experiment as in Fig. 1B and 1C. Numbers indicate %phospho-H3 positive cells. Red color indicates the S-phase cells with strong

γH2AX levels shown in Fig. 1B. (**d**) Scatter plots from flow cytometry analysis of chromatin bound CDC45 from an experiment as in Fig. 1D. Numbers indicate median CDC45 levels relative to mock in cells within the S-phase region (marked in green color).

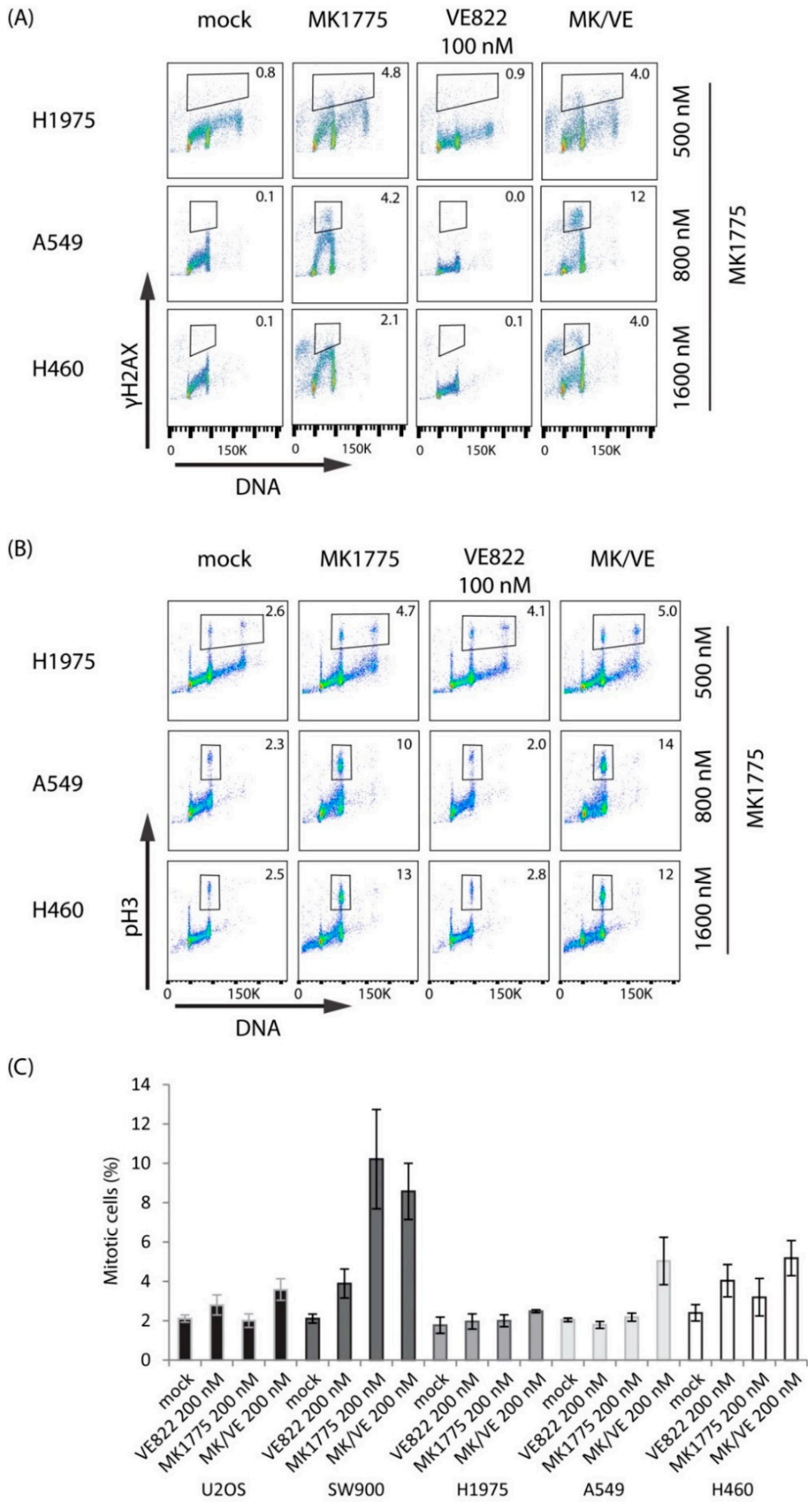

Figure S2. In lung cancer cell lines synergistic reduction in survival does not correlate with DNA damage induction in S-phase or with premature mitosis. (a) and (b) Flow cytometry analysis of H1975, A549 and H460 cells treated with high concentrations of MK1775 (500/800/1600 nM) and/or VE822 (100 nM) for 24 h or left untreated (mock). (a) Scatter plots of γH2AX versus DNA content (Hoechst). Numbers indicate % cells with strong γH2AX levels (within the marked region).

(b) Scatter plots of phospho-H3 versus DNA content. Numbers indicate % of cells in mitosis (within the marked region). (c) Percentage of cells in mitosis from experiments as in Figure 5A (n=3).

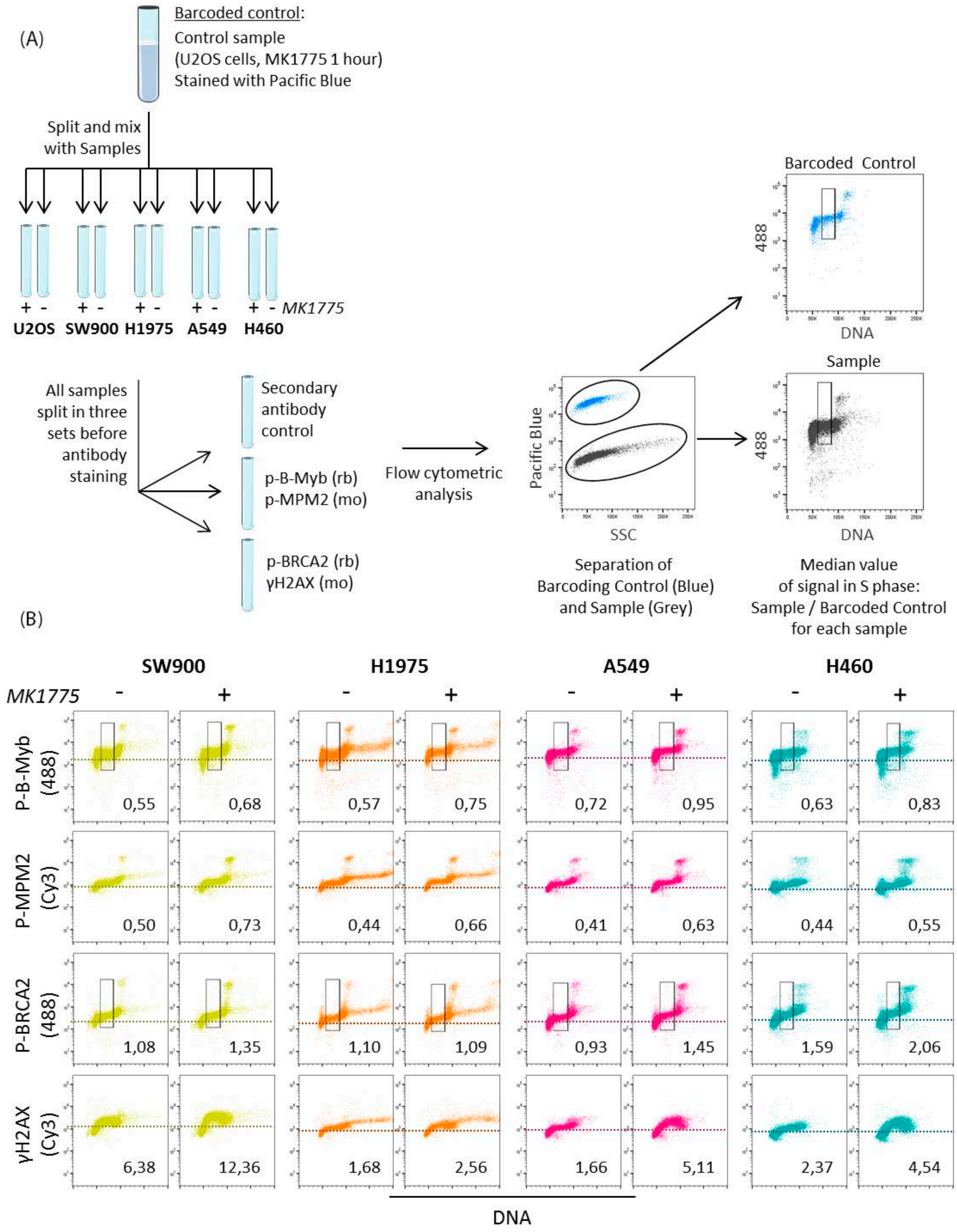

Figure S3. CDK-activity measurements in lung cancer cell lines. (a) Schematic overview of the flow cytometry-based CDK activity assay. A control sample (in this case U2OS cells treated as indicated) is stained with Pacific Blue, and divided between all samples of interest. The samples (which now include the sample and the barcoded control) are now split in three sets; One set is used for secondary antibody background control, the second set is stained with antibodies against phospho-B-Myb (rabbit (rb)) and phospho-MPM2 (mouse (mo)), and the third set is stained with antibodies against phos-pho-BRCA2 (rabbit) and γH2AX (mouse). All sets are then stained with secondary antibodies (anti-rabbit Alexa 488 and

anti-mouse Cy3) and FxCycleFarRed (DNA). Analysis is performed with a flow cytometer. Gating strategy: After single cell determination (not shown), the Pacific Blue signal is used to separate the barcoded control from the sample of interest. Then, the median values of the 488 and Cy3 signals are found both from the sample and the barcoded control (Example is shown for 488). This gating strategy is also performed in the secondary antibody control sets, and the signal values are subtracted from the samples and barcoding controls in the two other sets. Finally, the signal intensity of Alexa488 and Cy3 from the sample is divided on the signal intensity of the barcoded control, in all sets. In this way, sample-to-sample variations in staining are minimized, as the barcoded control sample is the same in all sets. (b) Raw data for experiment shown in Figure 3C. Staining and analysis was performed as described in A. Determined from the DNA stain, a region of S-phase cells was set. The Alexa 488- and Cy3-signals were measured from the same cells in each set (the Cy3 signals were measured from the same cells/in the same region as the 488 signals, therefore no regions are shown in the Cy3 dot plots). Values represent median signal intensity of the sample divided by the median signal intensity of the barcoded control. Background signals were also subtracted, as described in A.

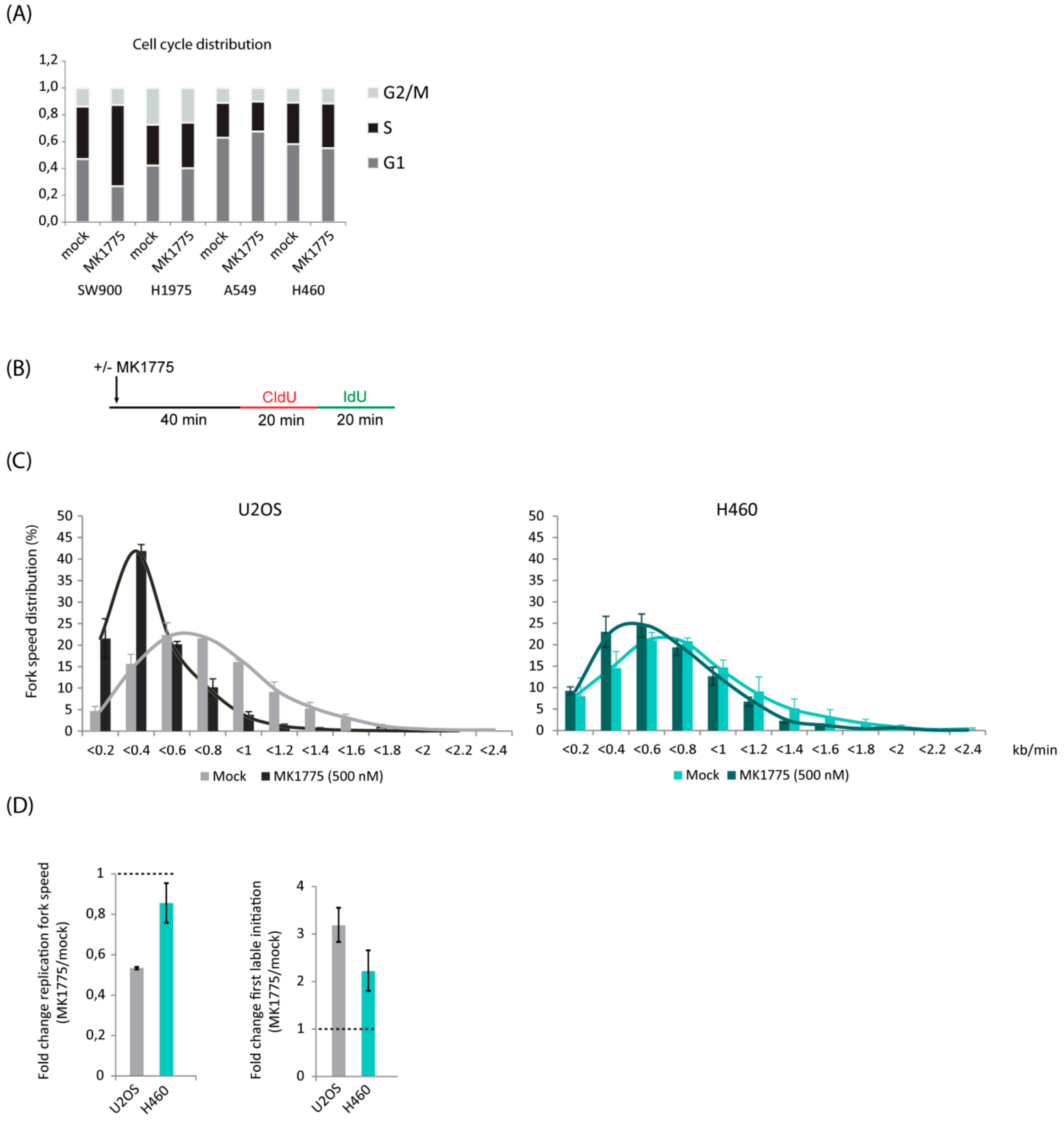

Figure S4. Resistant cell lines show no accumulation of cells in S-phase and continue replication in the presence of WEE1 inhibitor. (a) Cell cycle distribution of lung cancer cells comparing untreated cells (mock) to cells treated with 200 nM of MK1775 for 24 h. (b) Schematic presentation of experimental setup for DNA fiber analysis (c) Fork speed (kb/min) distribution in percentage from fiber analysis in U2OS and H460 cells with and without MK1775 (500 nM) treatment for 80 min.

(d) Left: average replication fork rate (kb/min). Error bars: SD (n=2). Right: first label origin firing (green-red-green) quantified as a percentage of all red (CldU-labelled) tracks. Error bars: SEM (n=3).

(A)

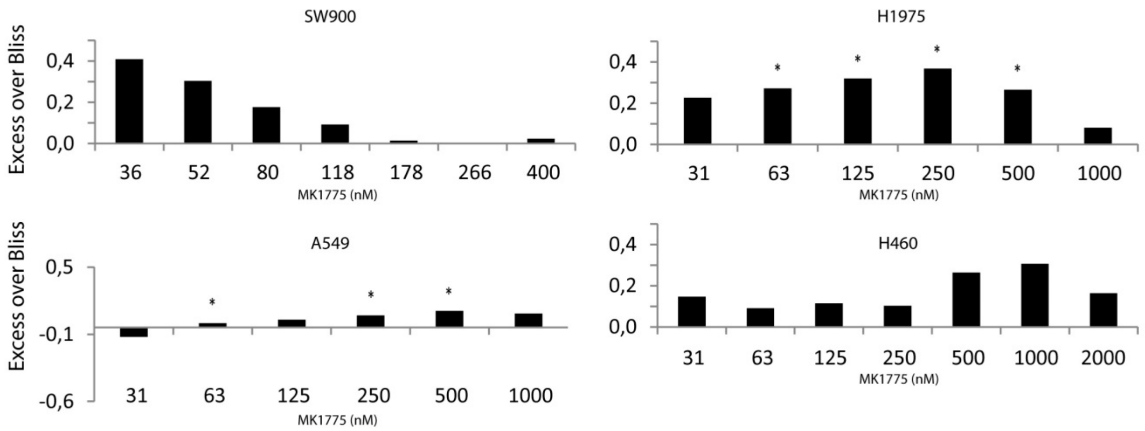

(B)

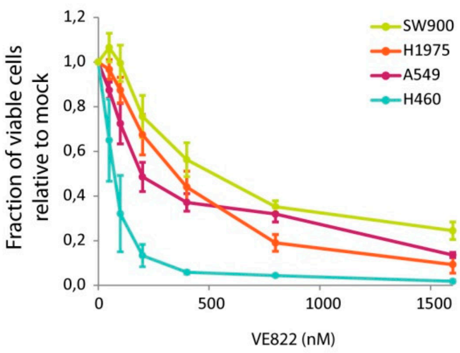

(C) Excess over Bliss

SW900

VE822 (nM) \ MK1775 (nM)	25	50	100	200	400
100	-0,04	0,12	0,14	0,09	-0,04
200	0,24	* 0,22	* 0,35	0,17	-0,04
400	0,14	* 0,16	* 0,22	0,09	*-0,03

A549

VE822 (nM) \ MK1775 (nM)	100	200	400	800	1600
50	0,06	0,22	0,06	0,04	0,03
100	0,25	0,20	0,16	0,04	0,01
200	* 0,16	* 0,19	0,07	-0,02	-0,24

H460

VE822 (nM) \ MK1775 (nM)	100	200	400	800	1600
25	* 0,15	0,25	0,23	* 0,09	* 0,02
50	* 0,18	* 0,32	* 0,19	0,10	0,14
100	* 0,34	* 0,42	* 0,41	* 0,20	* 0,02

Figure S5. Synergy scores and ATR inhibitor sensitivity in lung cancer cell lines. (a) Synergy scores (excess over Bliss) for combination treatments from figure 4A. (b) CellTiter-Glo viability assays in SW900, H1975, A549 and H460 cells treated with the indicated concentrations of VE822 for 24 hours and assayed at 4 days after drug removal. Error bars: SEM (n=3). (c) Table of synergy scores presented in Figure 4C. p values were determined by the two-tailed Student's one-sample t test, *p<0.05.

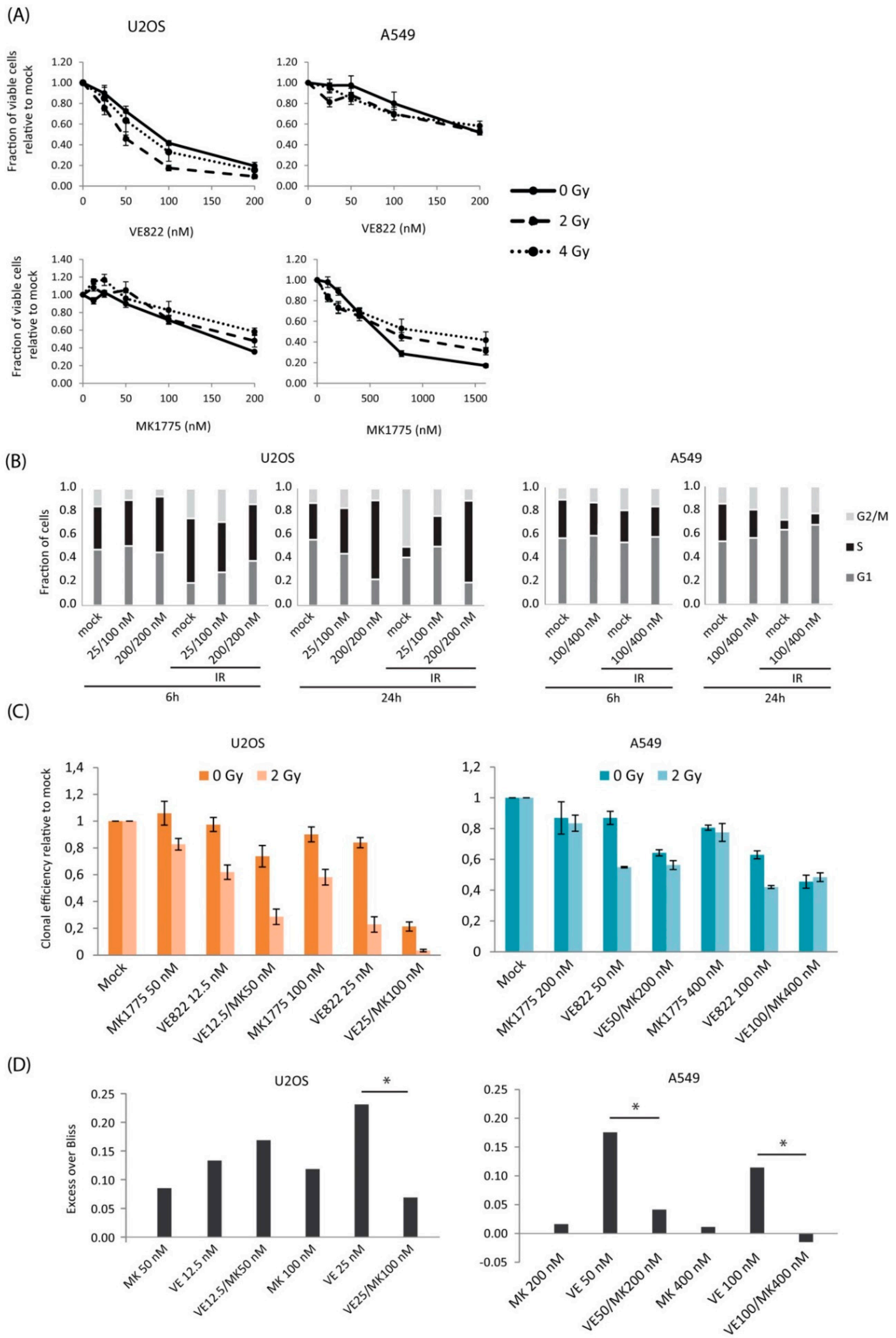

Figure S6. Radiosensitizing effect of WEE1 and ATR inhibitors in U2OS and A549. Data are from the same experiment as in Figure 6A. Fraction of viable cells relative to no-drug samples (for each radiation dose) are plotted, showing the effect of VE822 (upper panels) and MK1775 (lower panels). Error bars: SEM (n=4) (b) Cell cycle distribution of U2OS cells 6 and 24 h after combined treatment with VE822/MK1775 (25/100 and 200/200 nM for U2OS and 100/400 nM for A549) w/wo IR (4 Gy). (c) Data are from the same experiment as in Figure 6B, showing the fraction of colonies relative to mock. Results

are normalized to the values without drug at 0 Gy and 2 Gy, respectively. The average survival fraction at 2 Gy relative to 0 Gy was 0.4 for U2OS and 0.6 for A549 cells. (d) Synergy scores for inhibitors in combination with radiation (excess over Bliss) from the same data as in Figure 6B. *p<0.05 determined by the two-tailed Student's two-sample t-test.

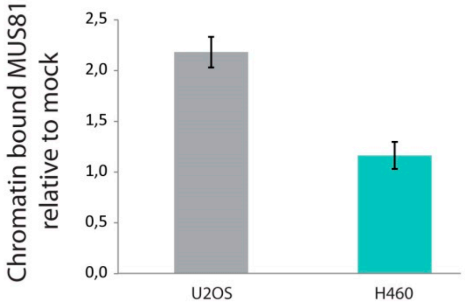

Figure S7. Chromatin loading of MUS81 is more increased in U2OS than in H460 in response to WEE1 inhibition. Flow cytometry analysis of chromatin bound MUS81 in H460 and U2OS cells treated for 6 h with 1 μM and 200 nM MK1775, respectively. Median levels were measured within a region of cells with S-phase DNA content similarly as in Figure S1D.

Table S1. Approximate IC50 values (MK1775 and VE822) in lung cancer cell lines based on CellTiter-Glo experiments.

	**Fig. 2A**	**Fig. 4A**	**Guertin et. Al**	**Fig. S4 B**
**Cell line**	**IC50, MK1775**	**IC50, MK1775 + 100 nM VE822**	**IC50, MK1775**	**IC50, VE822**
SW900	140 nM	120 nM	135 nM	540 nM
H1975	500 nM	125 nM	998 nM	330 nM
A549	1000 nM	750 nM	1859 nM	250 nM
H460	1700 nM	600 nM	3310 nM	70 nM

Uncropped western blots

Fig. 1A

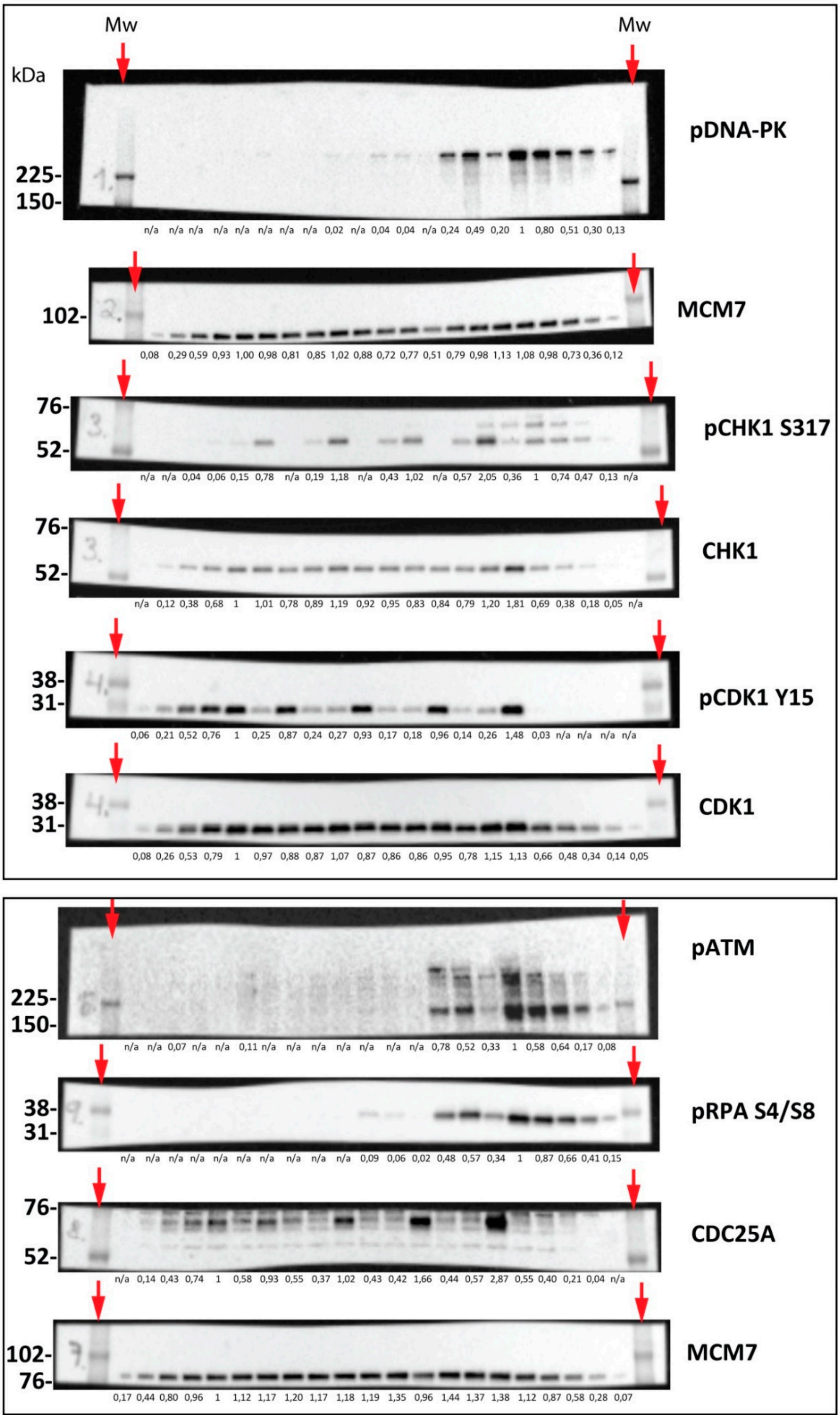

Fig. 1A

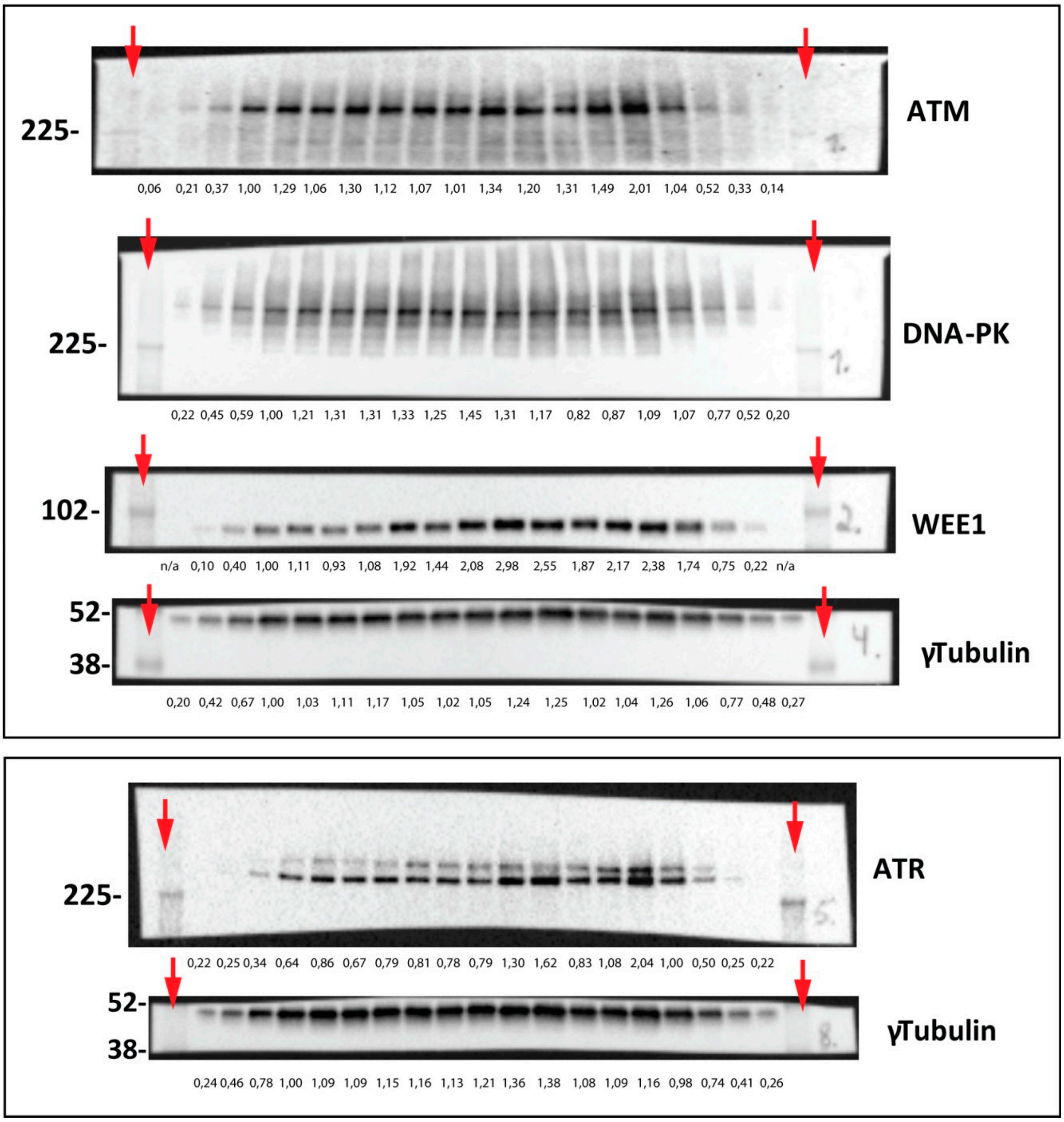

Blots shown in Fig. 1A.

Samples were run on four separate gels with γTubulin and MCM7 as loading controls. In main figure loading control (MCM7) is only shown from one gel. The four outermost lanes on each side show a dilution series (75, 50, 25 and 10 %) of mock (left) and 24h MK/VE (right). The dilution series are not included in main figure. Numbers show relative intensity of individual bands. Arrows indicate molecular weight marker (Mw) Rainbow Full Range (GERPN800E, VWR).

Fig. 2C

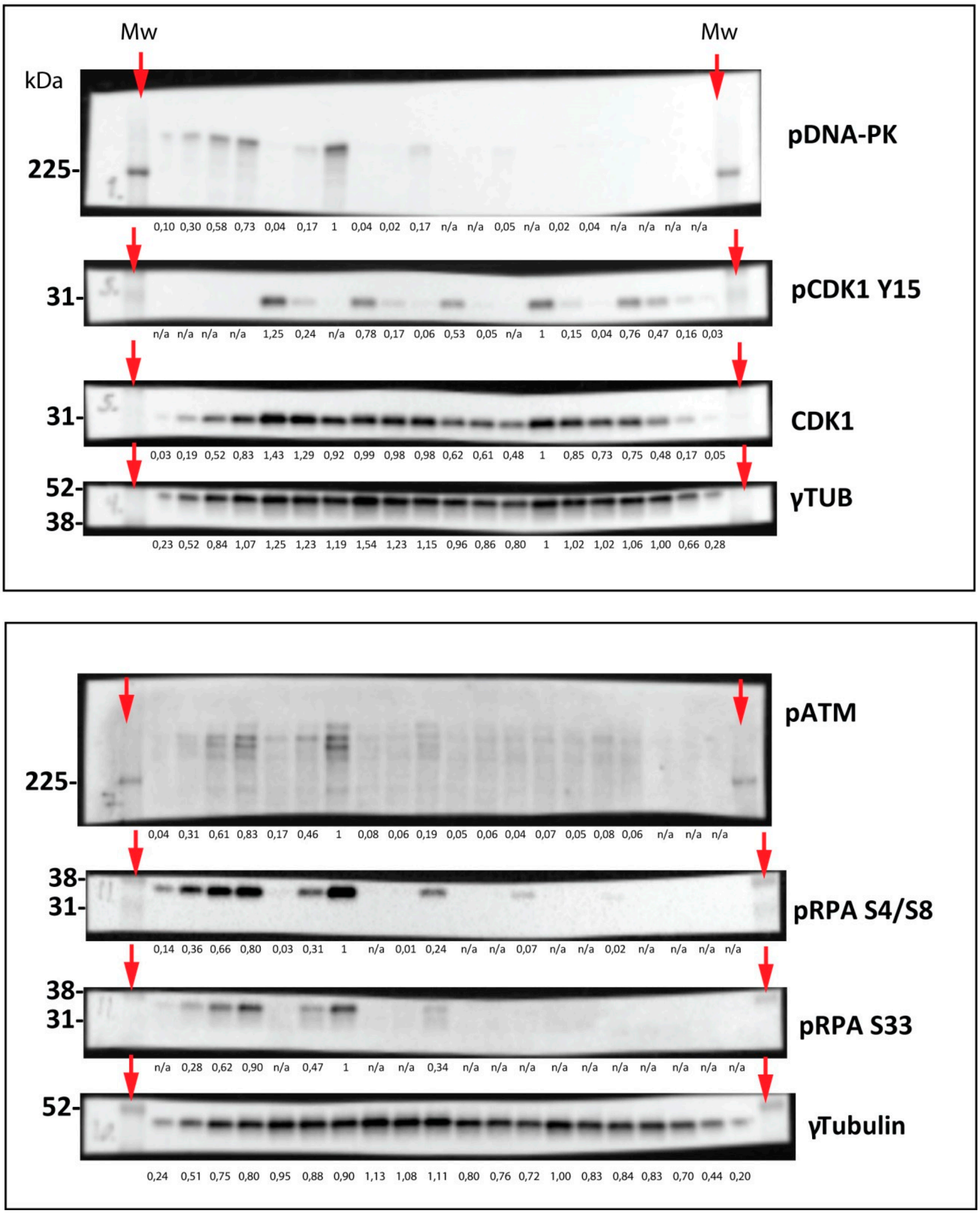
Mw
Mw
kDa
225-
pDNA-PK
0,10 0,30 0,58 0,73 0,04 0,17 1 0,04 0,02 0,17 n/a n/a 0,05 n/a 0,02 0,04 n/a n/a n/a n/a
31-
pCDK1 Y15
n/a n/a n/a n/a 1,25 0,24 n/a 0,78 0,17 0,06 0,53 0,05 n/a 1 0,15 0,04 0,76 0,47 0,16 0,03
31-
CDK1
0,03 0,19 0,52 0,83 1,43 1,29 0,92 0,99 0,98 0,98 0,62 0,61 0,48 1 0,85 0,73 0,75 0,48 0,17 0,05
52-
38-
γTUB
0,23 0,52 0,84 1,07 1,25 1,23 1,19 1,54 1,23 1,15 0,96 0,86 0,80 1 1,02 1,02 1,06 1,00 0,66 0,28
225-
pATM
0,04 0,31 0,61 0,83 0,17 0,46 1 0,08 0,06 0,19 0,05 0,06 0,04 0,07 0,05 0,08 0,06 n/a n/a n/a
38-
31-
pRPA S4/S8
0,14 0,36 0,66 0,80 0,03 0,31 1 n/a 0,01 0,24 n/a n/a 0,07 n/a n/a 0,02 n/a n/a n/a n/a
38-
31-
pRPA S33
n/a 0,28 0,62 0,90 n/a 0,47 1 n/a n/a 0,34 n/a n/a n/a n/a n/a n/a n/a n/a n/a n/a
52-
γTubulin
0,24 0,51 0,75 0,80 0,95 0,88 0,90 1,13 1,08 1,11 0,80 0,76 0,72 1,00 0,83 0,84 0,83 0,70 0,44 0,20

Fig. 2C

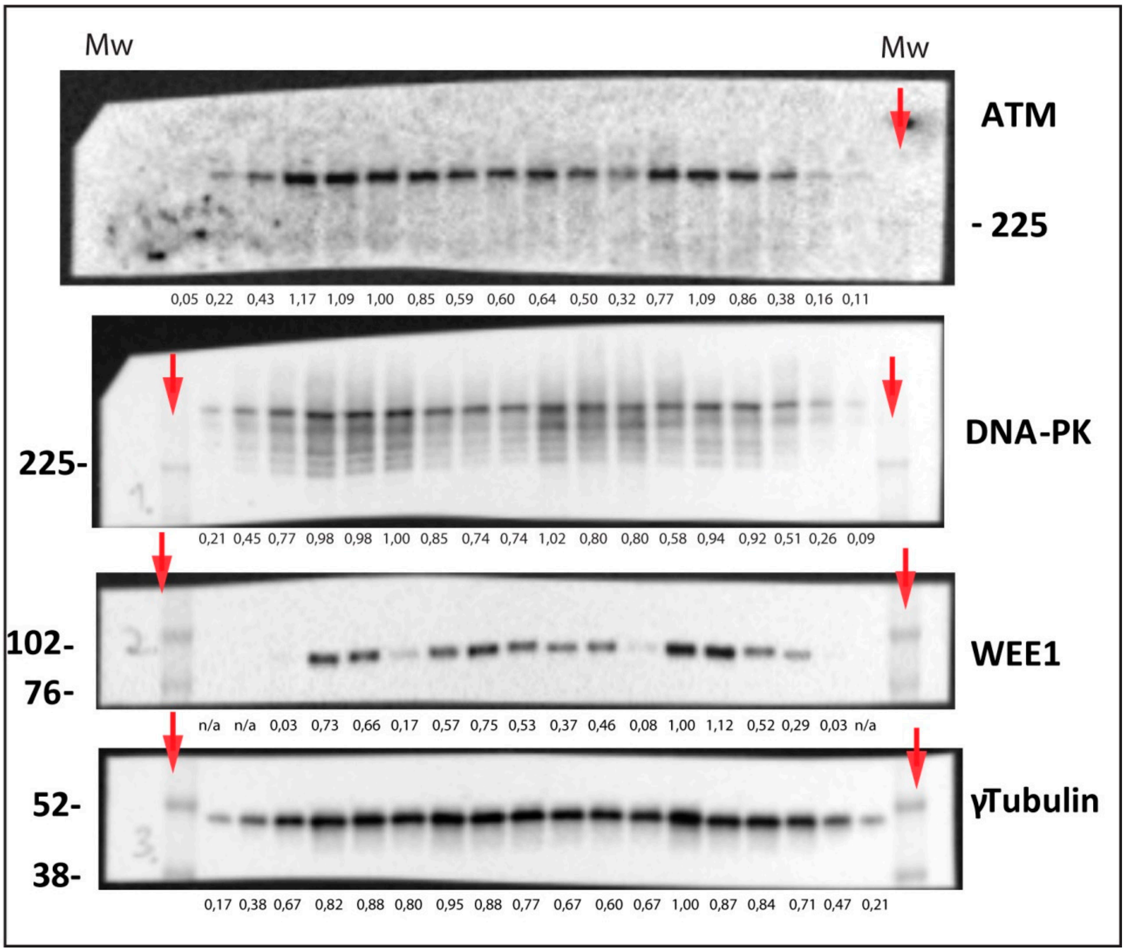

Blots shown in Fig. 2C.

The samples were run on four separate gels. Only one of the loading controls (γTubulin) are shown in Figure 2C. The four outermost lanes on each side show a dilution series (75, 50, 25 and 10 %) of SW900 treated with 1000 nM of MK1775 (left) and H460 mock (right). The dilution series are not included in main figure. Numbers show relative intensity of individual bands. Arrows indicate Rainbow Full Range molecular weight marker (Mw).

Fig. 3A_A549_H460

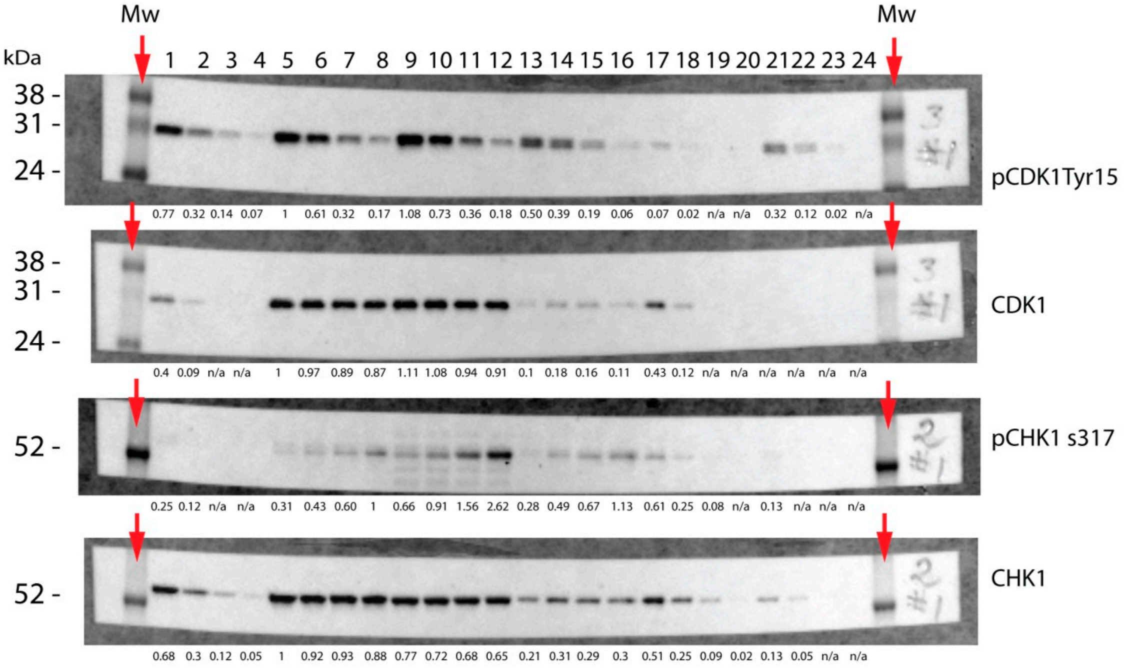

Fig. 3A_U2OS_H1975_SW900

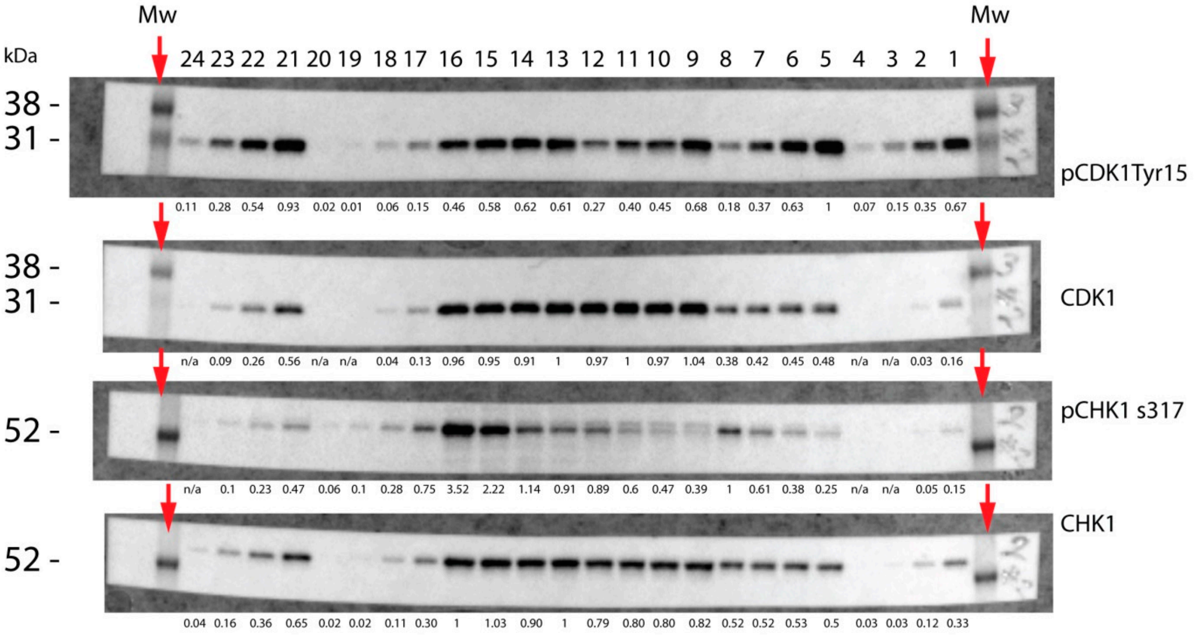

Blots shown in Figure 3A.

Upper: Lanes 5-12: A549 and H460 shown in main figure. Lanes 1-4: Dilution series (50, 25, 12.5 and 6.25%) of H460 mock. Lanes 17-20: Dilution series of H460 treated with 400 nM MK1775. Lanes 13-16 and 21-24: samples from an unrelated experiment. **Lower:** Lanes 5-8: U2OS treated as the other cell lines shown in main figure. Lanes 9-16: H1975 and SW900 shown in main figure. Lanes 1-4: Dilution series (50, 25, 12.5 and 6.25%) of U2OS mock. Lanes 17-20: Dilution series of

U2OS treated with 400 nM MK1775. Lanes 21-24: Dilution series of SW900 mock. (The blots are horizontally flipped compared to main figure.) Numbers below blots show relative intensity of individual bands. Arrows indicate Rainbow Full Range molecular weight marker (Mw).

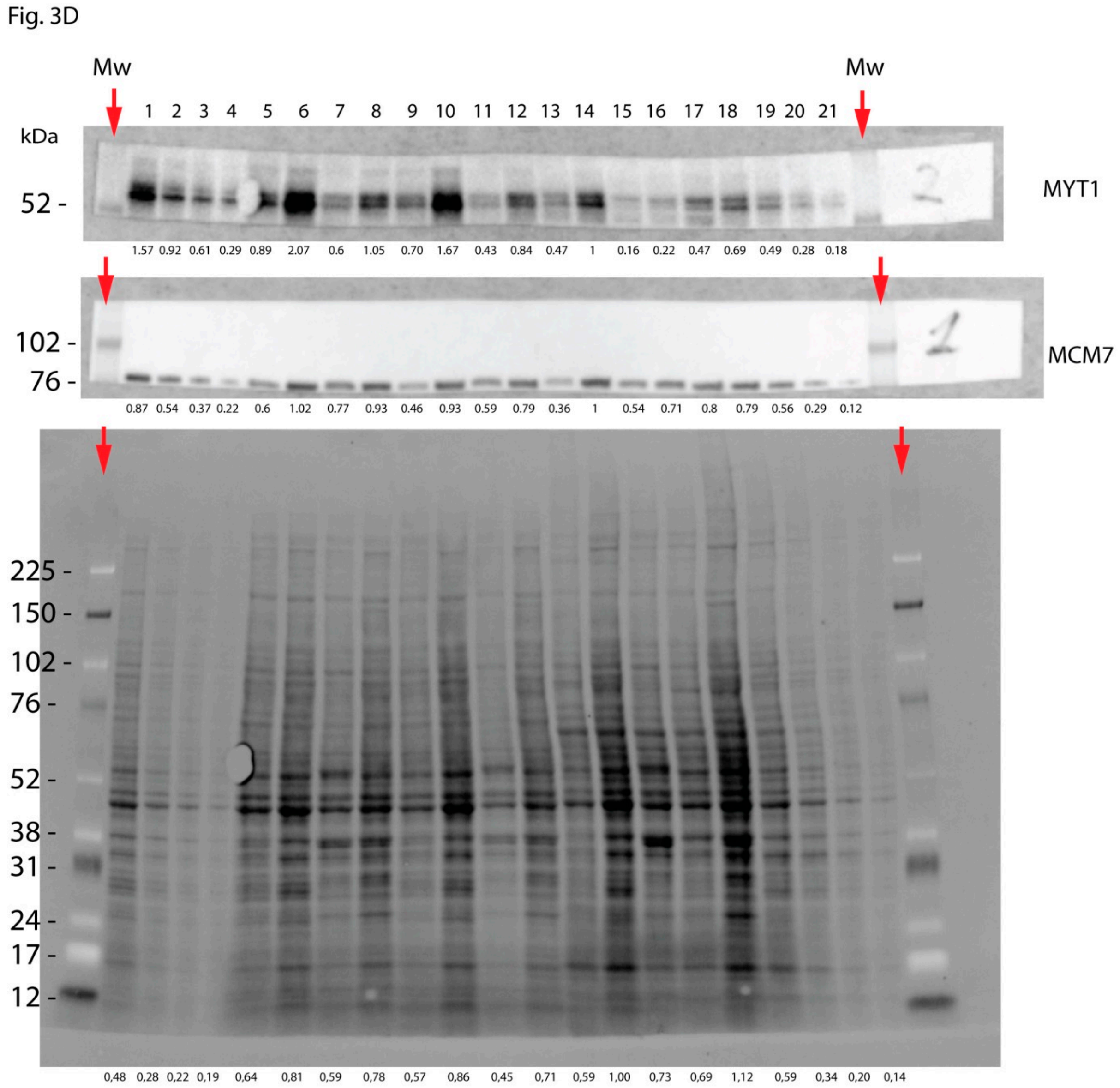

Blots shown in Fig. 3D.

Lanes 9-12: SW900, H1975, A549 and H460 shown in main figure. Lanes 1-4: Dilution series (50, 25, 12.5 and 6.25%) of sample in lane 6. Lanes 5, 6, 7 and 8: SW900, H1975, A549 and H460 from a separate sampling. Lanes 13-17: SW900, A549, U2OS and H460 from a separate sampling. Lanes 18-21: Dilution series (50, 25, 12.5 and 6.25%) of H1975 from lane 14. Lower: image of blot after transfer of stain-free gel showing total protein. Dilution series have been used for quantifications. Numbers below blots show relative intensity of individual bands. Arrows indicate Rainbow Full Range molecular weight marker (Mw).

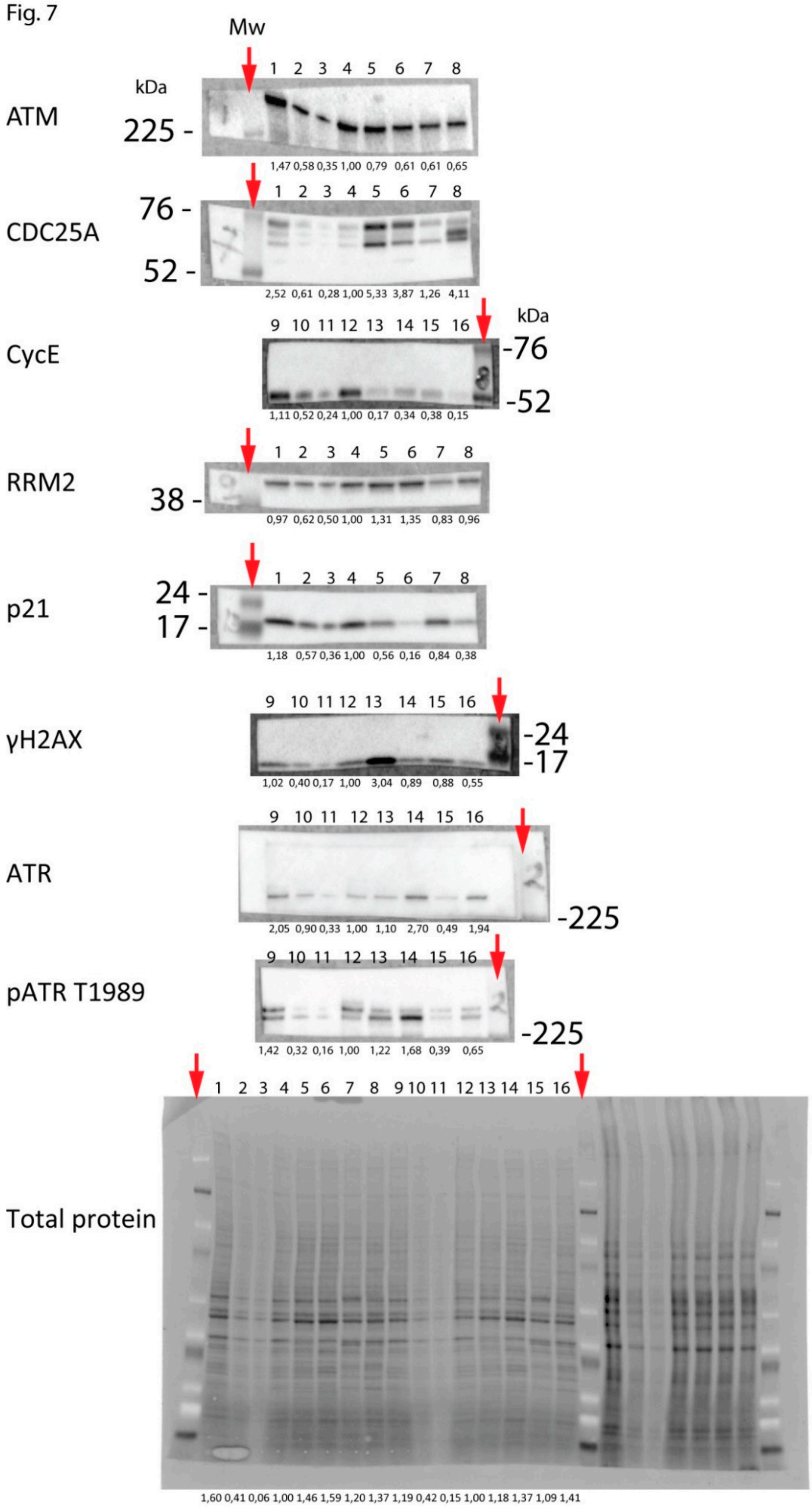

Blots shown in Figure 7.

Samples were run in parallel (lane 1-8 and lane 9-16), and membrane vertically cut in half. Lane 1-3 and 9-11: dilution series (125, 50 and 25 %) of samples in lanes 4 and 12. Lower: image of blot after transfer of stain-free gel showing total protein. Dilution series have been used for quantifications. Numbers below blots show relative intensity of individual bands. Arrows indicate Rainbow Full Range molecular weight marker (Mw).

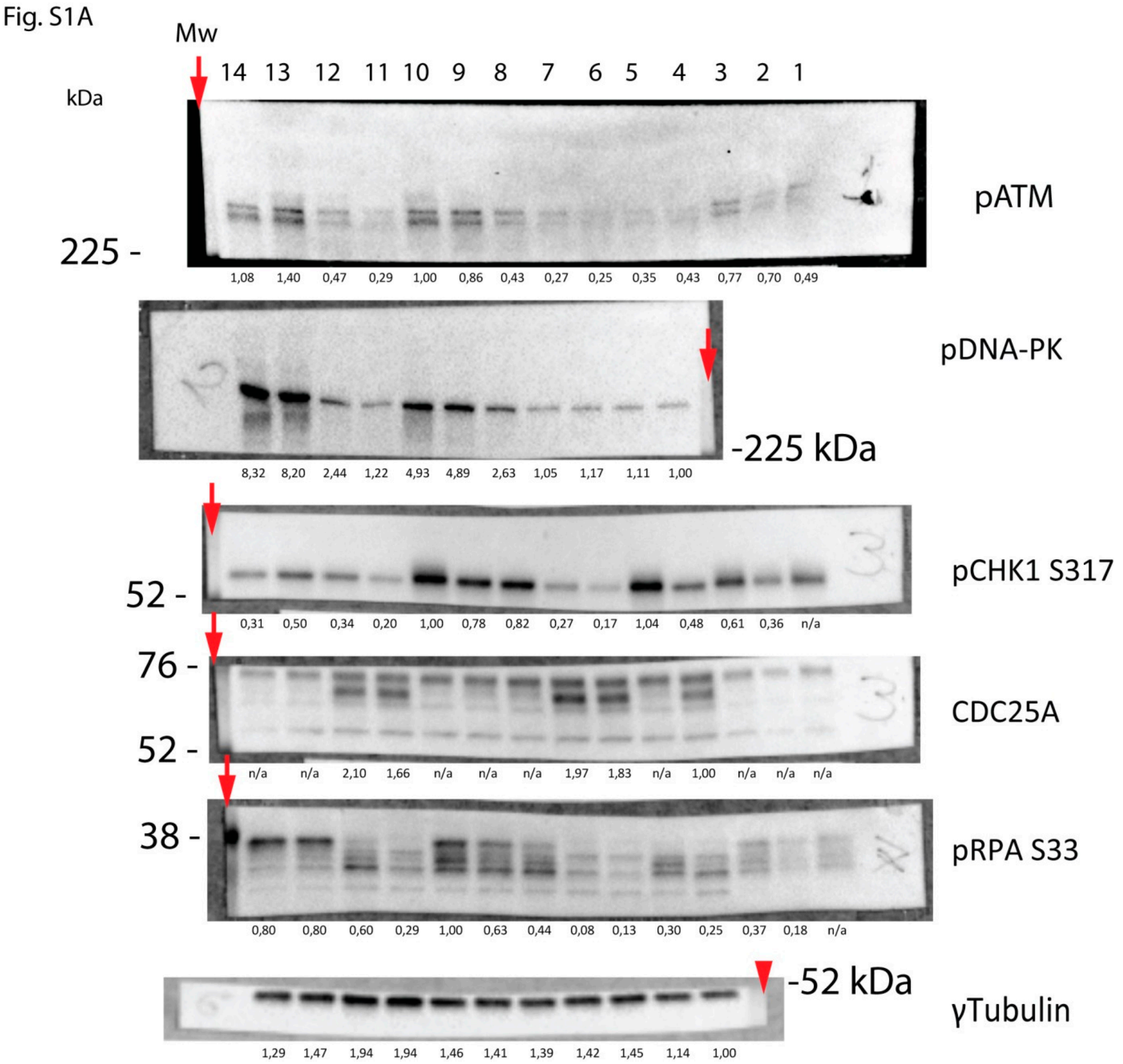

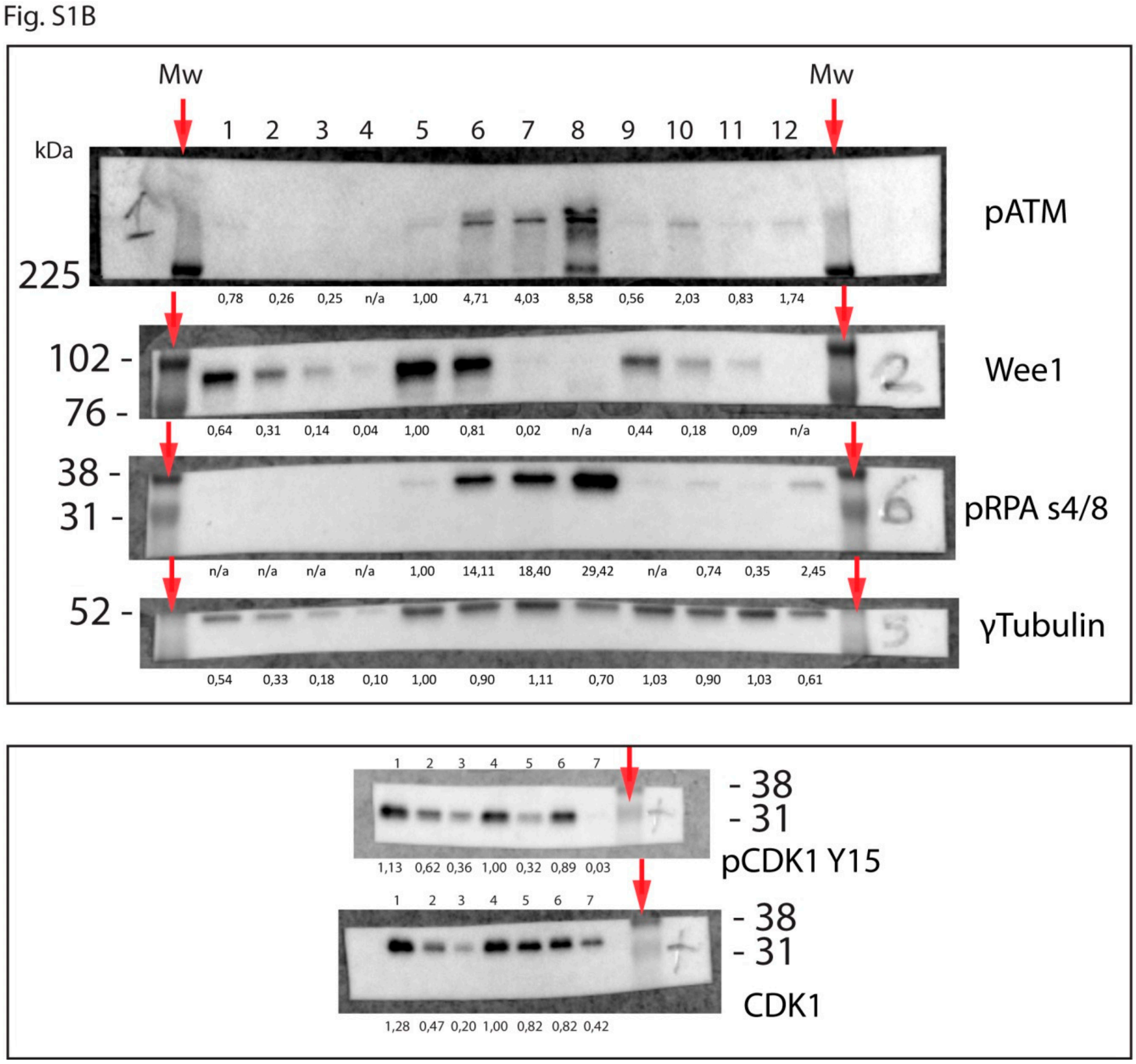

Blots shown in Fig. S1.

(a) Lanes 4-14 are shown above (Figure S1A). (The blot is horizontally flipped compared to S1A.) Samples were run on two gels. Lane 1: unrelated sample. Lane 2 and 3: dilution series (50 and 25%) of sample in lane 10 (dilution series was included in only one of the two gels). **(b)** Samples were run on two gels. Upper: Lanes 1-8 are shown above (Figure S1B). Lanes 9-12: Samples from an unrelated experiment. Lower: All lanes are shown above (Figure S1B). Numbers below blots show relative intensity of individual bands. Arrows indicate Rainbow Full Range molecular weight marker (Mw).
